# Diversity of mechanisms to control bacterial GTP homeostasis by the mutually exclusive binding of adenine and guanine nucleotides to IMP dehydrogenase

**DOI:** 10.1002/pro.4314

**Published:** 2022-04-27

**Authors:** David Fernández‐Justel, Íñigo Marcos‐Alcalde, Federico Abascal, Nerea Vidaña, Paulino Gómez‐Puertas, Alberto Jiménez, José L. Revuelta, Rubén M. Buey

**Affiliations:** ^1^ Metabolic Engineering Group, Department of Microbiology and Genetics Universidad de Salamanca Salamanca Spain; ^2^ Molecular Modeling Group, Centro de Biología Molecular Severo Ochoa CBMSO (CSIC‐UAM) Madrid Spain; ^3^ Biosciences Research Institute, School of Experimental Sciences Universidad Francisco de Vitoria Madrid Spain; ^4^ Wellcome Sanger Institute Hinxton UK

**Keywords:** (p)ppGpp, allosteric regulation, bacterial GTP homeostasis, IMP dehydrogenase, protein structure and function, purine nucleotide biosynthesis

## Abstract

IMP dehydrogenase(IMPDH) is an essential enzyme that catalyzes the rate‐limiting step in the guanine nucleotide pathway. In eukaryotic cells, GTP binding to the regulatory domain allosterically controls the activity of IMPDH by a mechanism that is fine‐tuned by post‐translational modifications and enzyme polymerization. Nonetheless, the mechanisms of regulation of IMPDH in bacterial cells remain unclear. Using biochemical, structural, and evolutionary analyses, we demonstrate that, in most bacterial phyla, (p)ppGpp compete with ATP to allosterically modulate IMPDH activity by binding to a, previously unrecognized, conserved high affinity pocket within the regulatory domain. This pocket was lost during the evolution of Proteobacteria, making their IMPDHs insensitive to these alarmones. Instead, most proteobacterial IMPDHs evolved to be directly modulated by the balance between ATP and GTP that compete for the same allosteric binding site. Altogether, we demonstrate that the activity of bacterial IMPDHs is allosterically modulated by a universally conserved nucleotide‐controlled conformational switch that has divergently evolved to adapt to the specific particularities of each organism. These results reconcile the reported data on the crosstalk between (p)ppGpp signaling and the guanine nucleotide biosynthetic pathway and reinforce the essential role of IMPDH allosteric regulation on bacterial GTP homeostasis.

## Abstract 2

PDB Code(s): 7PJI and 7PMZ;

## 1 INTRODUCTION

Purine nucleotides are essential molecules that cells synthetize in two different ways. In the de novo pathway, the purine ring system is stepwise assembled from 5‐phospho‐α‐d‐ribose 1‐diphosphate, while the *salvage* pathway recycles preformed nucleobases, nucleosides, and nucleotides.

**FIGURE 1:**
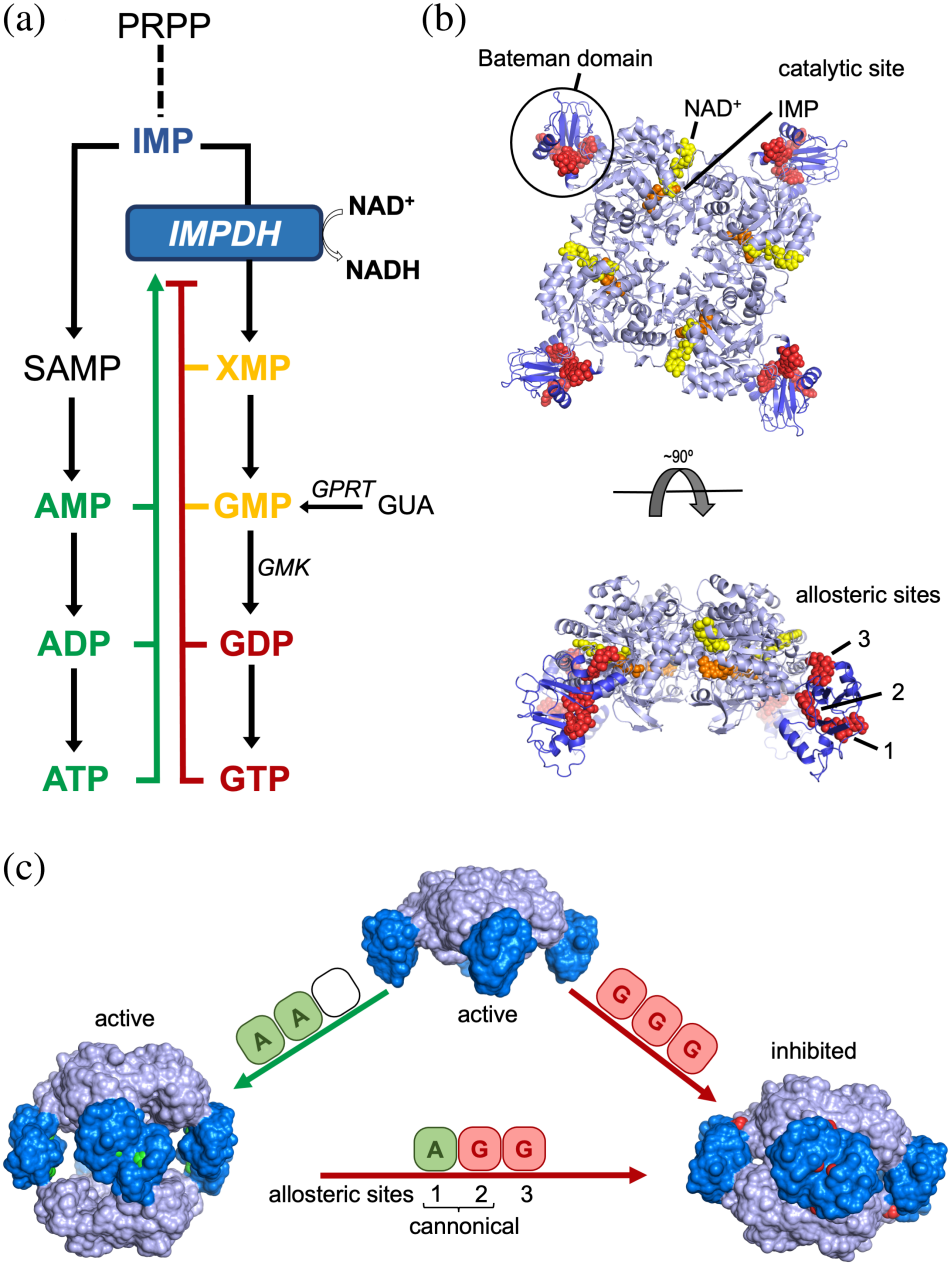
Structure, function, and regulation of eukaryotic IMPDHs. (a) Schematic and simplified scheme of the de novo purine nucleotide biosynthetic pathways. Competitive inhibitors are colored in yellow, while allosteric activators and inhibitors are colored in green and red, respectively. (b) Ribbon representation of an IMPDH tetramer, showing the catalytic domain (light blue) with the substrates NAD (yellow spheres) and IMP (orange spheres) and the regulatory Bateman domain (dark blue) with three GDP molecules (red spheres) bound. (c) Nucleotide binding to the allosteric sites in the Bateman domain promotes tetramer dimerization into octamers with different conformations and catalytic activities. IMPDH is represented as protein surface with the catalytic and regulatory domains light and dark blue colors, respectively. Adenine and guanine nucleotides bound to the Bateman regulatory domain are shown as spheres colored in green and red, respectively. IMPDH, IMP dehydrogenase

IMP dehydrogenase (IMPDH) catalyzes the first step in the guanine nucleotide de novo biosynthetic pathway, at the bifurcation of the guanine and adenine routes, which share the precursor IMP (Figure 1a). This constitutes a rate‐limiting step essential for balancing the metabolic flux through these parallel synthesis pathways. Therefore, IMPDH plays important roles in homeostasis maintenance and the inhibition of its catalytic activity has antiproliferative effects. Indeed, several drugs that target IMPDH are widely used at present for antiviral and immunosuppressive chemotherapy.^1^, ^2^, ^3^, ^4^ As an important pharmacological target, IMPDH has been object of various structural and functional studies that include the identification of a large variety of inhibitors.^5^ Nonetheless, the physiological regulation of IMPDH remain unclear and it has only been since the past few years that we are starting to envisage the diversity and complexity of its regulatory mechanisms.^6^, ^7^, ^8^, ^9^, ^10^, ^11^, ^12^, ^13^, ^14^, ^15^

The basic units of IMPDH are tetramers that dimerize to form octamers upon nucleotide binding. An IMPDH monomer consists of a catalytic TIM barrel (Figure 1b; light blue) and a regulatory Bateman domain (Figure 1b; dark blue), which is not required for catalytic activity but is essential for allosteric regulation. GMP^16^ and XMP^17^ have been reported as competitive inhibitors of IMPDH in vitro (Figure 1a) although it remains unclear if this has relevance in vivo since these molecules are not strong inhibitors even at concentrations that are 10‐fold greater than physiological.^7^, ^18^

Eukaryotic IMPDHs contains three allosteric sites (Figure 1b) that operate coordinately to modulate the catalytic activity. Sites 1 and 2 are canonical cystathionine beta synthase motifs, conserved among Bateman domains,^19^ that bind either adenine (ATP/ADP/AMP) or guanine (GTP/GDP) nucleotides. The third allosteric noncanonical site, exclusive of eukaryotic IMPDHs, can only bind the guanine nucleotides GTP or GDP.^6^ The binding of adenine nucleotides to the canonical Sites 1 and 2 induces extended active octamers, while binding of guanine nucleotides to the allosteric Sites 2 and 3 induces compact octamers (Figure 1c). Octamer compaction forces the active sites of opposing tetramers to interact, forming an interdigitated pseudo beta‐barrel that disfavors substrate binding and inhibits catalytic activity. The disruption of any of the three allosteric sites generate constitutively activated mutants^7^ and several missense mutations mapping into these sites have been associated to severe retinopathies^20^, ^21^ and dystonia.^22^

The mechanism of IMPDH allosteric regulation is fine tuned in eukaryotic cells through post‐translational modifications, such as phosphorylation,^23^, ^24^ as well as protein polymerization into mesoscale polymers denoted as *rod and rings* or *cytoophidia*.^25^, ^26^, ^27^, ^28^ Phosphorylation and polymerization desensitize IMPDH to GTP/GDP‐mediated inhibition and are triggered when the cell needs a boost of GTP, for example, in conditions of high‐rate growth or in response to light during the visual cycle in retinal photoreceptors.^8^, ^13^, ^14^, ^23^, ^25^

In bacteria, the IMPDH enzyme is encoded by the essential gene *guaB* (we will use IMPDH to refer indistinctly to bacterial and eukaryotic enzymes). In contrast to the eukaryotic enzymes, bacterial IMPDHs only contains two canonical allosteric binding sites in their Bateman domains. Bacterial IMPDHs have been previously reported to be insensitive to guanine nucleotide allosteric inhibition.^7^, ^10^ In turn, the catalytic activity of some bacterial IMPDHs is modulated by the binding of ATP to the Bateman domain.^10^ According to this observation, Munier–Lehmann's group proposed a classification for bacterial IMPDHs. *Class I* IMPDHs form inhibited compact octamers in vitro that switch to extended active octamers upon ATP binding, while *Class II* IMPDHs are active tetramers that shift to extended (also active) octamers in the presence of ATP.^12^

Increasing experimental evidence point to a relevant role of Bateman domains of IMPDHs in GTP homeostasis. In *Escherichia coli*, the regulatory domain is essential to maintain the intracellular ATP/GTP balance within a narrow physiological range.^18^, ^29^ In *Bacillus subtilis*, mutations within the Bateman domain of IMPDH suppress the characteristic phenotype of (p)ppGpp deficiency, suggesting a functional connection between IMPDH allosteric regulation and alarmone signaling.^30^, ^31^, ^32^ Nonetheless, to our knowledge, no physiological mechanism of allosteric inhibition of bacterial IMPDHs has been reported.

In this study, we unveil the diversity of molecular mechanisms of allosteric regulation of bacterial IMPDHs and describe their structural and biochemical basis. These data explain the differences found on the regulation of the guanine nucleotide biosynthesis among bacterial phyla and allow us to propose their plausible evolutionary trajectory. Most possibly, the bacterial IMPDH ancestor was allosterically modulated by the mutually exclusive binding of ATP and (p)ppGpp to the Bateman domain of IMPDH. (p)ppGpp occupy a previously unrecognized site that partially overlaps with the canonical Site 2, where ATP also binds. During the evolution of the proteobacterial lineage, this site was lost and, in turn, the Bateman domain of the IMPDH from most Proteobacteria evolved to be directly modulated by the balance between ATP and GTP, which compete for the canonical Site 2.

In this way, high ATP/GTP—or ATP/(p)ppGpp—ratios favor an extended, catalytically active, conformation. In contrast, when these ratios decrease, guanine nucleotide binding to the regulatory domain induces a compact conformation that significantly reduce the catalytic activity. Thereby, the adenine/guanine nucleotide balance controls a conformational switch that closely resembles that reported for the eukaryotic enzymes, demonstrating the universality of this mechanism. Moreover, in line with eukaryotic enzymes, our data suggest that bacterial IMPDHs also fine‐tune the conformational switch by post‐translational modifications, such as lysine acetylation. Altogether, these observations represent an excellent example of how evolution has generated different versions of the same mechanism of regulation to adapt to the specific metabolic requirements of each organism. Furthermore, given the therapeutic value of IMPDH, the results presented in this manuscript might have important implications for drug design and boost novel therapeutic approaches.

## 2 RESULTS

### 2.1 In the presence of ATP, GTP, and GDP significantly inhibit the IMPDH of *E. coli* and *Pseudomonas aeruginosa*

We and others have reported that guanine nucleotides by themselves are not able to significantly inhibit the activity of bacterial IMPDHs in vitro, presumably because they lack the third noncanonical site, which is exclusive of eukaryotic enzymes.^7^, ^10^ Nonetheless, we revisited this issue bearing in mind that intracellular levels of ATP are usually significantly higher than GTP.^33^ Thereby, we tested the effects of guanine nucleotides on the activity of preformed ATP‐induced octamers in vitro.

**FIGURE 2:**
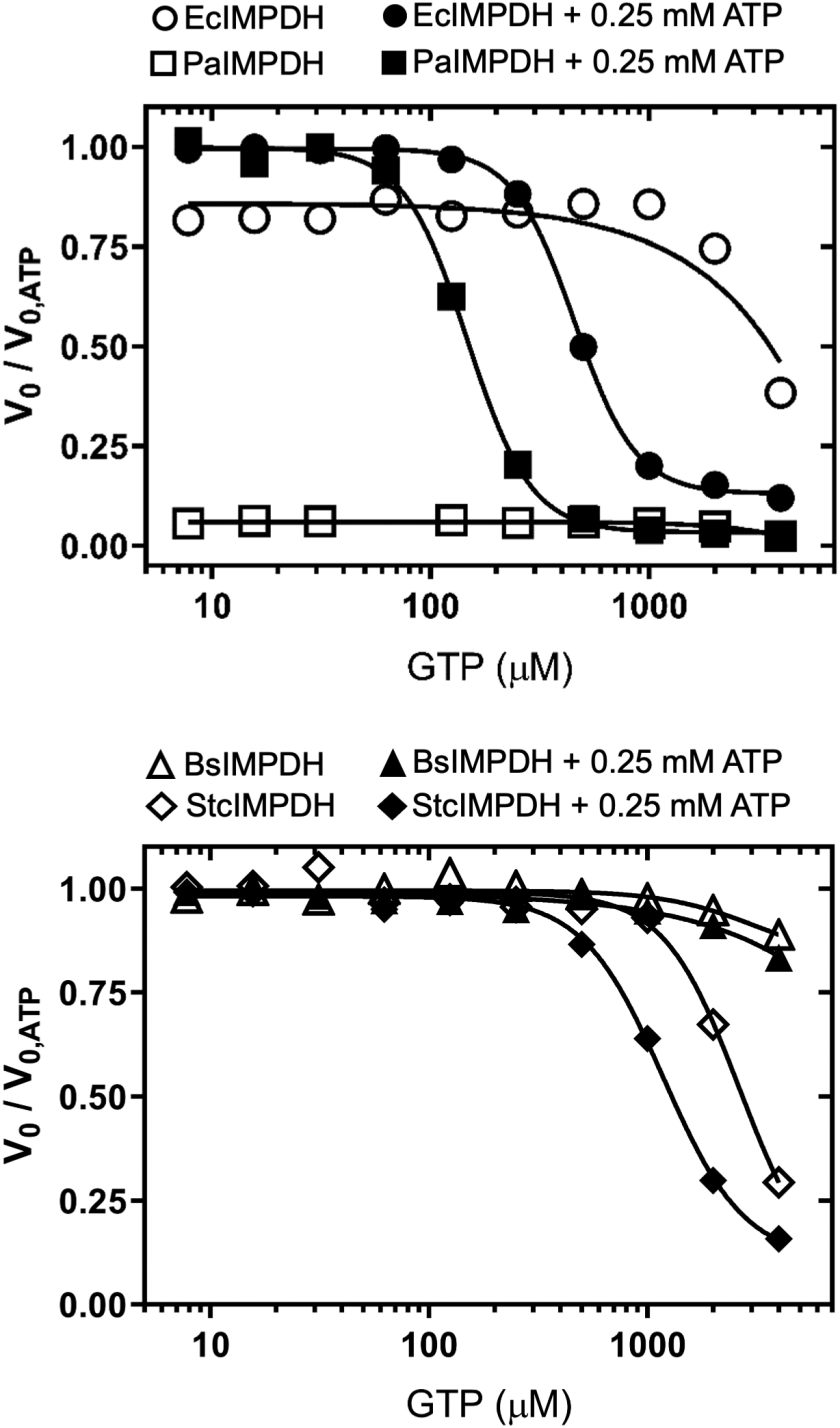
Effects of guanine nucleotides on the catalytic activity of IMPDH in vitro. Graphs showing the normalized initial velocity values (*V*_0_ values in the absence of GTP divided by the respective values in the presence of GTP). The *V*_0_ values used for the normalization of the data are EcIMPDH 16.6 ± 1.4, PaIMPDH 26.0 ± 0.7, BsIMPDH 14.2 ± 0.7, and StcIMPDH 14.0 ± 0.3 nM s^−1^ (mean ± std. error). Estimated IC_50_ values for are 455.3 ± 6.3 μM and 147.4 ± 3.9 μM (mean ± std. error) for EcIMPDH and PaIMPDH, respectively. Similar results were obtained for GDP inhibition (Figure S1). BsIMPDH, *Bacillus subtilis* IMPDH; EcIMPDH, *Escherichia coli* IMPDH; IMPDH, IMP dehydrogenase; PaIMPDH, *Pseudomonas aeruginosa* IMPDH; StcIMPDH, *Streptomyces coelicolor* IMPDH

Corroborating previous reports^7^, ^10^ that GTP/GDP alone did not have a significant effect on the catalytic activity of the four bacterial IMPDHs assayed in vitro (Figures 2 and SS1). In contrast, when 0.25 mM ATP is present in the solution, GTP/GDP could readily inhibit the enzymes from the γ‐Proteobacteria *E. coli* and *P. aeruginosa* (EcIMPDH and PaIMPDH, respectively), with *K*_i,app_ values in the mid‐micromolar range (Figure 2). On the other side, GTP/GDP could only very weakly inhibit the enzymes from the Firmicute *B. subtilis* and the Actinobacteria *Streptomyces coelicolor* (BsIMPDH and StcIMPDH, respectively), with *K*_i,app_ values in the millimolar range (Figures 2 and SS1).

### 2.2 Crystallographic structures of *P. aeruginosa* bound to ATP and GDP

**FIGURE 3:**
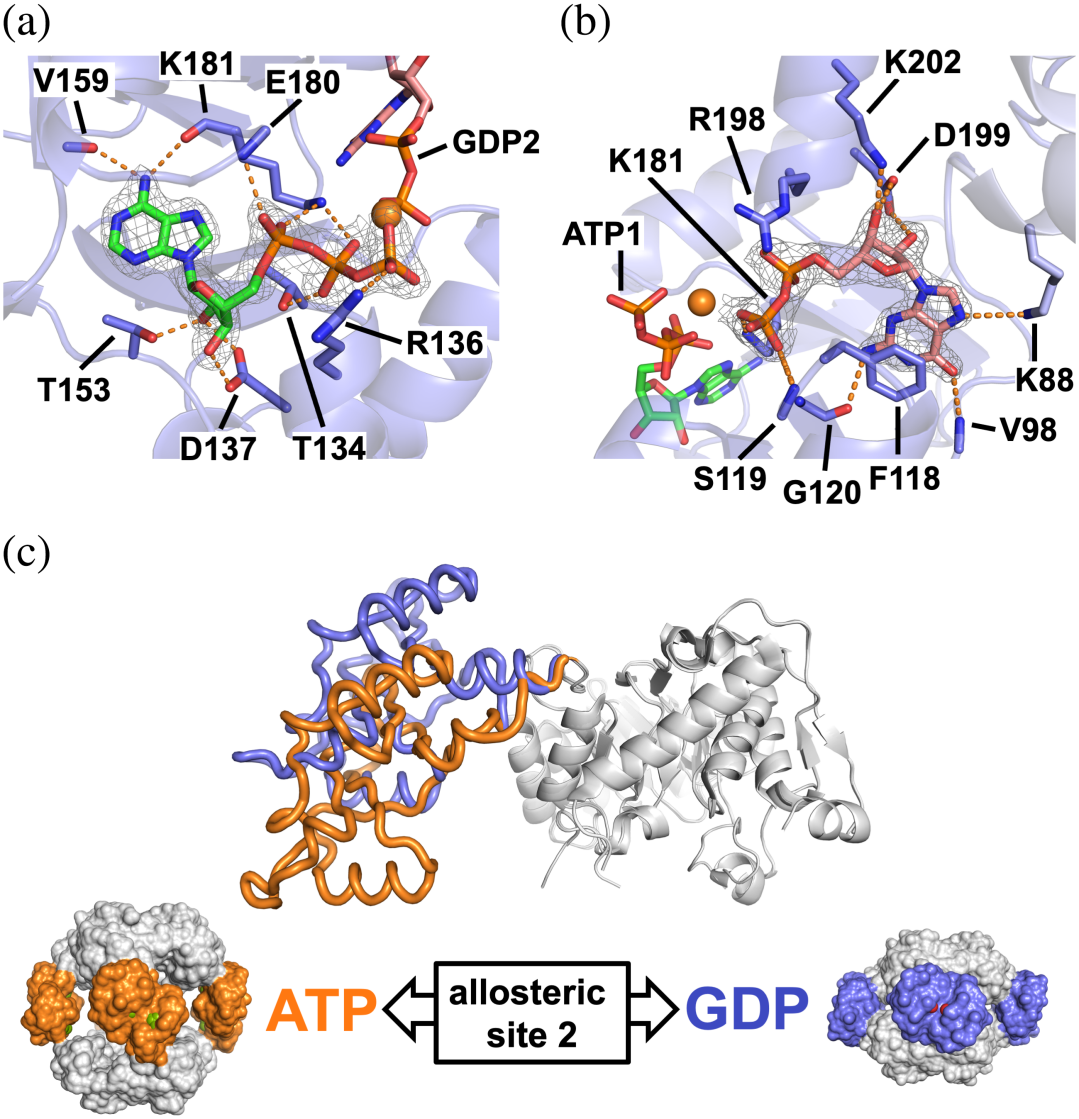
Structure of PaIMPDH bound to ATP and GDP. Detailed views of ATP (a) and GDP (b) bound in the Bateman domain to the first and second nucleotide canonical sites, respectively. IMPDH protein is represented in semitransparent blue cartoons with the side chain of key interacting residues shown in sticks. The 2mF_o_–DF_c_ electron density map, contoured at the 1.6σ level, is shown as a grey mesh. Key protein–nucleotide atomic interactions are represented as orange dashed lines and the coordinated Magnesium atom is shown as an orange sphere. (c) Upper panel: structural superposition of the catalytic domains (white ribbons) of a monomer of PaIMPDH showing the different conformations adopted by the Bateman domain upon ATP (orange ribbons; PDB ID 4DQW)^10^ or ATP/GDP (blue ribbons) binding. Lower panel: the conformational switch described in the upper panel, translated to the octameric structures. PaIMPDH octamers are represented as protein surfaces with the same color code as in the upper panel. IMPDH, IMP dehydrogenase; PaIMPDH, *Pseudomonas aeruginosa* IMPDH

To gain further insights into the molecular mechanisms of inhibition of GTP/GDP in proteobacterial IMPDHs and to map their binding sites, we aimed at obtaining the high‐resolution crystallographic 3D structures of enzyme–nucleotide complexes. After multiple cocrystallization trials, we were able to obtain the structure of PaIMPDH bound to both ATP and GDP at 1.65 Å resolution (Table SS1). The two monomers in the asymmetric unit (AU) contained well‐defined electron density in the Bateman domain that could be unequivocally attributed to ATP and GDP bound to the canonical Sites 1 and 2, respectively, as well as a magnesium atom coordinated by their β‐ and γ‐phosphates (Figure 3a, b). The binding modes of ATP and GDP in the canonical sites are identical to those observed in the structures of eukaryotic IMPDHs,^6^, ^13^, ^14^, ^34^ where the nucleotide's phosphate groups position close together at the interface of two opposing Bateman domains (Figure S2b).

The recognition of the adenine ring of ATP bound to the canonical Site 1 (ATP1) was mainly due to hydrogen bonds from the backbone carbonyl atoms of residues V159 and K181 and N6 nitrogen atom of the adenine. The O2 and O3 hydroxyl groups of the ribose moiety hydrogen bonded to the side chain of the absolutely conserved aspartic acid D137, as well as residue T153. ATP1 phosphate groups interacted with the basic side chains of residues R136 and K181. Additionally, ATP1 γ‐phosphate coordinated a Magnesium atom, together with the β‐phosphate of GDP bound to the canonical Site 2 (GDP2) and the carboxylic acid in the side chain of residue E180 (Figure 3a). In the canonical Site 2, GDP2 guanine ring was sandwiched between the hydrophobic side chains of residues F118 and V94, with the hydroxyls of the ribose moiety tightly coordinated to the carboxylic acid of the absolutely conserved residue D199.^35^ The negative charge of GDP2 phosphate groups was counteracted by the basic side chains of residues K181 and R198, as well as a Magnesium atom, as described above (Figure 3b). Mutations in any of the conserved Aspartic residues that define the canonical Sites 1 and 2 in EcIMPDH (D138N and D200N, which correspond to D137and D199 in PaIMPDH), abrogate GTP/GDP‐dependent allosteric inhibition (Figure S3a). These data further demonstrate the specificity of the interaction of these nucleotides in the Site 2 and the necessity of ATP bound to Site 1 for the inhibition.

Monomers in the AU are related by noncrystallographic symmetry axes that allow to reconstruct IMPDH octamers within the crystal lattice. These octamers are assembled as dimers of tetramers that pile up tail‐to‐tail, forcing the finger domains of opposing tetramers to interact and placing their catalytic sites close together (Figure S2b) to inhibit the catalytic activity. The comparison of the crystallographic structures of PaIMPDH‐ATP1/GDP2 (this work) and PaIMPDH‐ATP1/ATP2 (PDB code 4DQW)^10^ allows the identification of a conformational switch, which is controlled by the competition between adenine and guanine nucleotides for the allosteric Site 2 in the Bateman domain (Figure 3c). Small angle X‐ray scattering (SAXS) experiments further corroborate that the conformations observed in the crystal structures reliably represent those occurring in solution (Figure S4a). Remarkably, this conformational switch is essentially identical to the previously reported for eukaryotic IMPDHs.^6^, ^7^, ^8^, ^13^, ^14^, ^34^ Thereby, these data indicate that the purine nucleotide‐controlled conformational switch that modulates the activity of IMPDH is universally conserved form bacteria to eukaryotes.

Remarkably, no electron density surrounding the area corresponding to the eukaryotic noncanonical Site 3 is observed in the inhibited PaIMPDH structure, suggesting that, in contrast to eukaryotic IMPDHs, the occupancy of the canonical Site 2 by GTP/GDP (when ATP is bound at the canonical Site 1) is necessary and sufficient to induce compact octamers and, subsequently, inhibit the activity of PaIMPDH and EcIMPDH. To corroborate this hypothesis, we performed computational targeted molecular dynamics (TMD) simulations of monomers of PaIMPDH bound to different nucleotides. These simulations induce conformational changes by applying an external force to minimize the root mean square deviation between initial and final (target) structures, thus driving the molecule to the target conformation during the simulation. As shown in Figure S5a,b, when both canonical sites are occupied by ATP, PaIMPDH can easily oscillate between the active (extended) and inhibited (compacted) conformations, since the applied external force (and the subsequent accumulated work) needed to drive these changes (both extension and compaction) is very low. On the other hand, when GDP occupies Site 2, an increasing supply of energy is needed to activate (extend) the inhibited conformation but essentially no work is needed for the opposite change (Figure S5a,b). These results indicate that GDP binding to Site 2 strongly stabilizes PaIMPDH into the inhibited compacted conformation.

### 2.3 ATP/GTP balance allosterically modulates the activity of PaIMPDH and EcIMPDH

**FIGURE 4:**
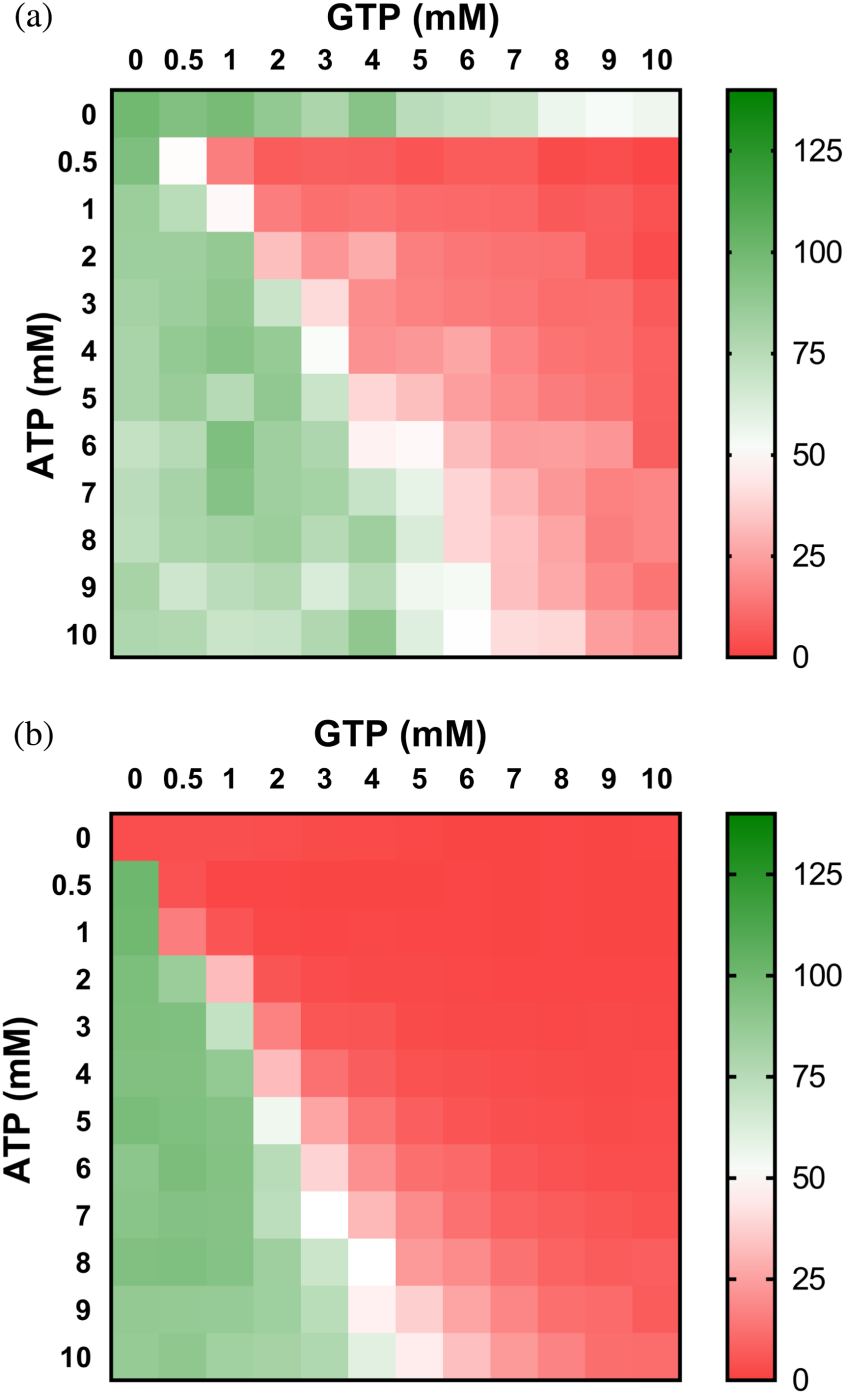
The ATP/GTP balance modulates the activity of proteobacterial IMPDHs. Heatmap representation of the enzymatic percent activity. *V*_0_ values at different ATP versus GTP concentrations, normalized to the *V*_0_ values in the absence of nucleotide for EcIMPDH (a) and at 1 mM ATP for PaIMPDH (b). The *V*_0_ values used for normalization are EcIMPDH 15.9 and PaIMPDH 16.4 nM s^−1^ (note that PaIMPDH is inactive in vitro in the absence of ATP^10^). IMPDH, IMP dehydrogenase; PaIMPDH, *Pseudomonas aeruginosa* IMPDH

The results shown above clearly indicate that the binding of adenine and guanine nucleotides in the second canonical site is mutually exclusive and, thereby, the balance between the concentration of these nucleotides will presumably determine the activity of the enzyme. We tested this hypothesis by assaying the effects of different concentrations of ATP and GTP in vitro on proteobacterial IMPDHs at IMP and NAD^+^ concentrations within the expected physiological levels.^18^, ^33^, ^36^ Figure 4 clearly shows how ATP and GTP compete to modulate the activity of IMPDH. Remarkably, EcIMPDH (panel a) and PaIMPDH (panel b) showed significant differences in nucleotide affinities, in accordance with the different IC_50_ values estimated from Figure 2.

At constant 3 mM ATP, which is in the expected range of intracellular levels in *E. coli* cells exponentially growing in minimal media,^18^ 1.3 and 2.3 mM GTP concentrations are needed to duplicate and raise 10‐fold the Km values of EcIMPDH, respectively (Figure S6). These GTP concentrations are easily reached in exponentially growing *E. coli* cells, and can be even higher upon addition of purine nucleobases and nucleosides to the culture media.^18^ Similarly, 0.8 and 1.3 mM GTP is required to duplicate and raise 10‐fold the *K*_m_ values of PaIMPDH (Figure S6). Altogether, these data indicate that the intracellular ATP/GTP ratio modulates proteobacterial IMPDH activity.

### 2.4 (p)ppGpp potently inhibit the catalytic activity of *B. subtilis* but has no effect on *E. coli* IMPDH

**FIGURE 5:**
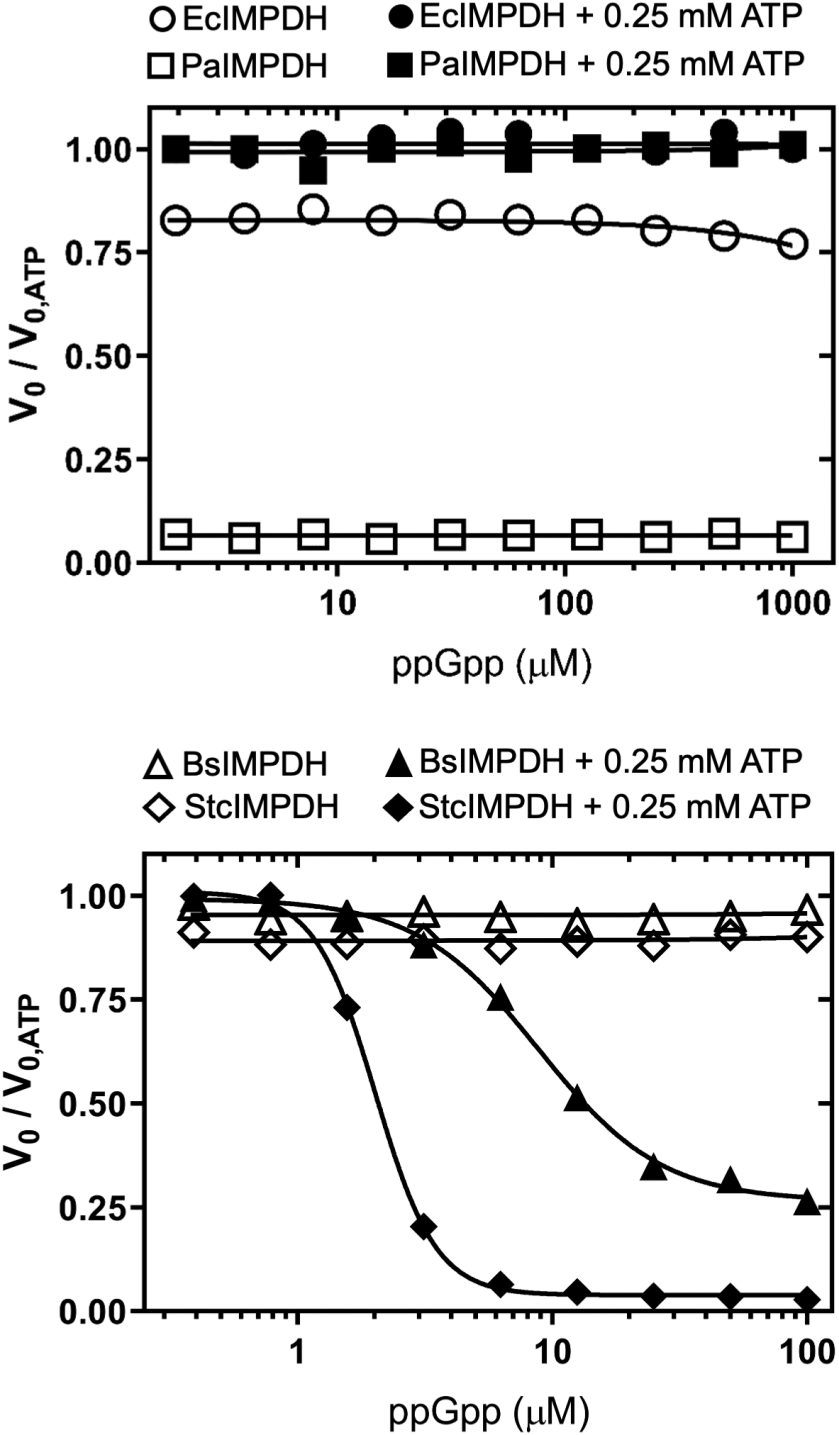
Effects of ppGpp on the catalytic activity of IMPDH in vitro. Graphs showing the normalized initial velocity values (*V*_0_ values in the absence of ppGpp divided by the respective values in the presence of ppGpp. The *V*_0_ values used for the normalization of the data are EcIMPDH 18.5 ± 1.0, PaIMPDH 26.7 ± 0.9, BsIMPDH 12.9 ± 0.8, and StcIMPDH 12.6 ± 0.4 nM s^−1^ (mean ± std. error). Estimated IC_50_ values are 8.9 ± 0.4 μM and 2.0 ± 0.03 μM (mean ± std. error) for BsIMPDH and StcIMPDH, respectively. Similar results were obtained for pppGpp inhibition (Figure S1). BsIMPDH, *Bacillus subtilis* IMPDH; EcIMPDH, *Escherichia coli* IMPDH; IMPDH, IMP dehydrogenase; PaIMPDH, *Pseudomonas aeruginosa* IMPDH; StcIMPDH, *Streptomyces coelicolor* IMPDH

As described in Section 1, it seems evident that bacterial IMPDHs must play a relevant role on (p)ppGpp signaling in vivo, despite the scarce information available and the reported differences among organisms.^30^, ^31^, ^32^, ^37^, ^38^, ^39^, ^40^ Prompted by this, we assayed the effects of (p)ppGpp on the activity of IMPDH in vitro in the presence or absence of ATP. As shown in Figure 5, ppGpp by itself has no significant effect on the catalytic activity in vitro of any of the enzymes assayed. In contrast, when combined with ATP, ppGpp can potently inhibit (in the low micromolar range) BsIMPDH and StcIMPDH (Figure 5). Similar results were obtained for pppGpp (Figure SS1). These data clearly indicate that in the presence of ATP, (p)ppGpp can inhibit these enzymes even at basal concentrations.^41^ In contrast, (p)ppGpp had no detectable effect in vitro on the activity of EcIMPDH or PaIMPDH, even at millimolar concentrations and independently on the presence or absence of ATP (Figures 5 and SS1).

### 2.5 Crystallographic structure of *S. coelicolor* IMPDH complexed to ATP and ppGpp

We then set cocrystallization experiments to obtain high‐resolution structures of (p)ppGpp‐IMPDH complexes and were able to solve the structure of the IMPDH from *S. coelicolor* bound to ATP and ppGpp at 2.0 Å resolution (Table SS1). The AU contained 16 IMPDH monomers that are related by symmetry axes and allow the reconstruction of IMPDH octamers within the crystal lattice. These octamers are formed by dimers of tetramers assembled with a conformation that resembles, with only minor deviations, those adopted in the presence of ATP and GDP by the proteobacterial PaIMPDH enzyme (Figure S7). SAXS experiments further corroborate that the conformation observed in the crystal structure matches that found in solution in the presence of ATP and ppGpp (Figure S4b). Moreover, in the presence of ATP alone, StcIMPDH adopts a conformation similar to PaIMPDH‐ATP (PDB ID 4DQW), highlighting the universality of the purine nucleotide‐controlled conformational switch.

**FIGURE 6:**
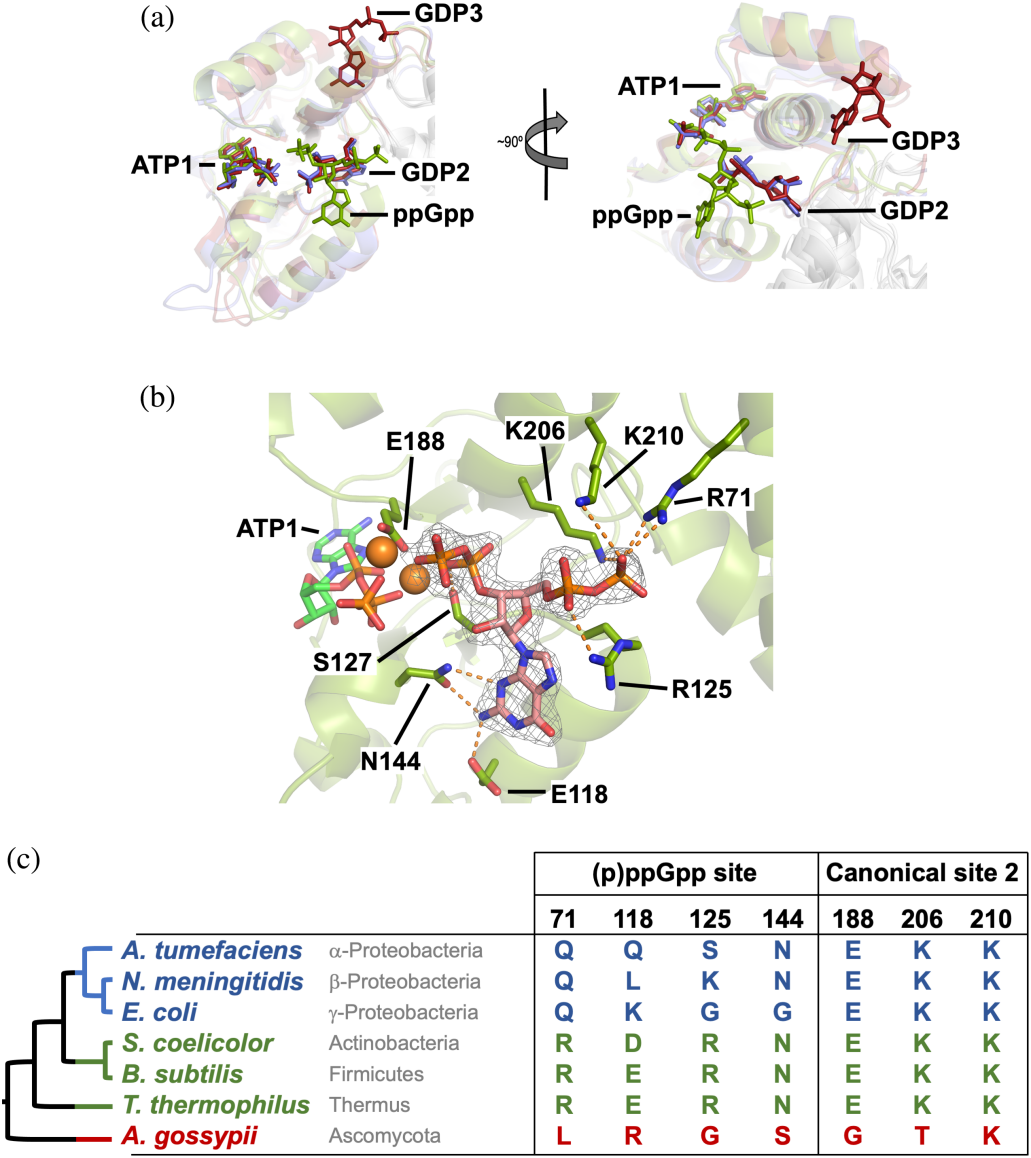
Structure of StcIMPDH bound to ATP and ppGpp. (a) Structural superimposition of the Bateman domains of PaIMPDH‐ATP/GDP (blue), StcIMPDH‐ATP‐ppGpp (green), and AgIMPDH‐ATP/GDP (red; PDB ID 5TC3).^6^ (b) Detailed view of the ppGpp binding site in the Bateman domain. IMPDH protein is represented in semitransparent green cartoons with the side chain of key interacting residues shown in sticks. The 2mF_o_–DF_c_ electron density map, contoured at the 1.6σ level, is shown as a grey mesh. Key protein–nucleotide atomic interactions are represented as orange dashed lines and the coordinated Magnesium atoms are shown as orange spheres. (c) The taxonomic distribution of the (p)ppGpp binding site within the Bateman domain is shown. The phylogenetic tree on the left shows the evolutionary relationships among the groups of bacteria (color‐coded according to a) and is extracted from a more detailed analysis shown in Figures S9 and S10. IMPDH, IMP dehydrogenase; PaIMPDH, *Pseudomonas aeruginosa* IMPDH; StcIMPDH, *Streptomyces coelicolor* IMPDH

All monomers in the AU showed well‐defined electron density in the Bateman domain that could be unequivocally attributed to ATP, ppGpp, and two magnesium atoms. ATP was found in the first canonical site (ATP1) with a binding mode identical to that observed in other IMPDH structures (5TC3, 4DQW, 5MCP, 6U8N, and 7RES).^6^, ^10^, ^14^, ^34^ Surprisingly, ppGpp was bound to a previously unrecognized pocket within the Bateman domain adopting an elongated T shape conformation.^42^ The (p)ppGpp binding site is different from either the second canonical (GDP2) or the third eukaryotic noncanonical site, although its δ‐ and ε‐phosphates partially occupy the canonical Site 2 (Figure 6a).

The ppGpp binding pocket in StcIMPDH is mostly formed by the polar side chains of residues E118, R125, and N144, that form hydrogen bonds with different atoms of the guanine ring. The α‐ and β‐phosphates tightly interact with the basic sidechain of residues, R71, R125, K206, and K210, whereas δ‐ and ε‐phosphates, which point toward the γ‐phosphate of ATP1, coordinate two Magnesium atoms, together with the carboxylic acid of residue E188 (Figure 6b). BsIMPDH mutant enzymes with the most relevant (p)ppGpp interacting residues substituted by their equivalents in EcIMPDH showed significantly reduced inhibition with respect to the wild‐type enzyme in vitro (Figure S3b). These data demonstrates that the newly discovered (p)ppGpp pocket of StcIMPDH is not artifactual but functional and conserved between *S. coelicolor* and *B. subtilis*. Furthermore, our mutational analysis also revealed that the absolutely conserved Aspartic residues that define the two canonical nucleotide binding sites in Bateman domains are also required for (p)ppGpp mediated inhibition (Figure S3b).

The analysis of a bacterial IMPDH multiple sequence alignment revealed that the (p)ppGpp binding site is conserved among most bacterial phyla but is consistently absent in α‐β‐γ‐Proteobacteria and some δ‐Proteobacteria genera (Figures 6c and S8). This observation perfectly explains why EcIMPDH and PaIMPDH cannot be inhibited by (p)ppGpp (Figures 5 and S1). The resulting phylogenetic tree (best model LG + G + I^43^) suggests that the allosteric regulation of IMPDH by (p)ppGpp is the ancestral state in bacteria and that its loss occurred during the evolution of Proteobacteria (Figure S9). However, the low bootstrap values of this tree prevented drawing solid conclusions. To obtain further support for this hypothesis, we also reconstructed a species tree for the set of bacteria under study, using multiple conserved proteins data obtained from a reference phylogeny database.^44^ The resulting phylogenetic tree (best model LG + G + F) perfectly agrees with the well stablished tree of life^45^ and resembles the IMPDH tree (Figure S10). Altogether, these data strongly support the hypothesis that the bacterial ancestral IMPDH was regulated by (p)ppGpp and this regulation was lost during the evolution of Proteobacteria.

No electron density was found in the canonical Site 2 or the noncanonical Site 3. Nonetheless, the results described above indicate that, in the presence of ATP bound to the canonical Site 1, the occupancy of the (p)ppGpp pocket is necessary and sufficient to induce the inhibited conformation. We then performed computational TMD simulations to corroborate this hypothesis. When the two canonical sites were occupied by ATP, StcIMPDH readily oscillates between the active (extended) and inhibited (compacted) conformations. In contrast, the occupancy of the (p)ppGpp pocket, in the presence of ATP1, implies a large amount of accumulated work to activate (extend) the inhibited conformation (Figure S5c and d). These results indicate that the occupancy of the (p)ppGpp binding pocket in StcIMPDH strongly stabilizes the inhibited compact conformation, similar to the binding of GTP/GDP to PaIMPDH (Figure S5a and b).

Altogether, these results further demonstrate that the allosteric control of the catalytic activity of IMPDH is mediated by a universal purine nucleotide‐controlled conformational switch. They also illustrate how evolution has diverged to adapt this regulatory mechanism to the specific particularities of each organism through the invention of different nucleotide‐binding pockets within the Bateman domain.

### 2.6 The ratio ATP/(p)ppGpp allosterically controls the activity of IMPDH

**FIGURE 7:**
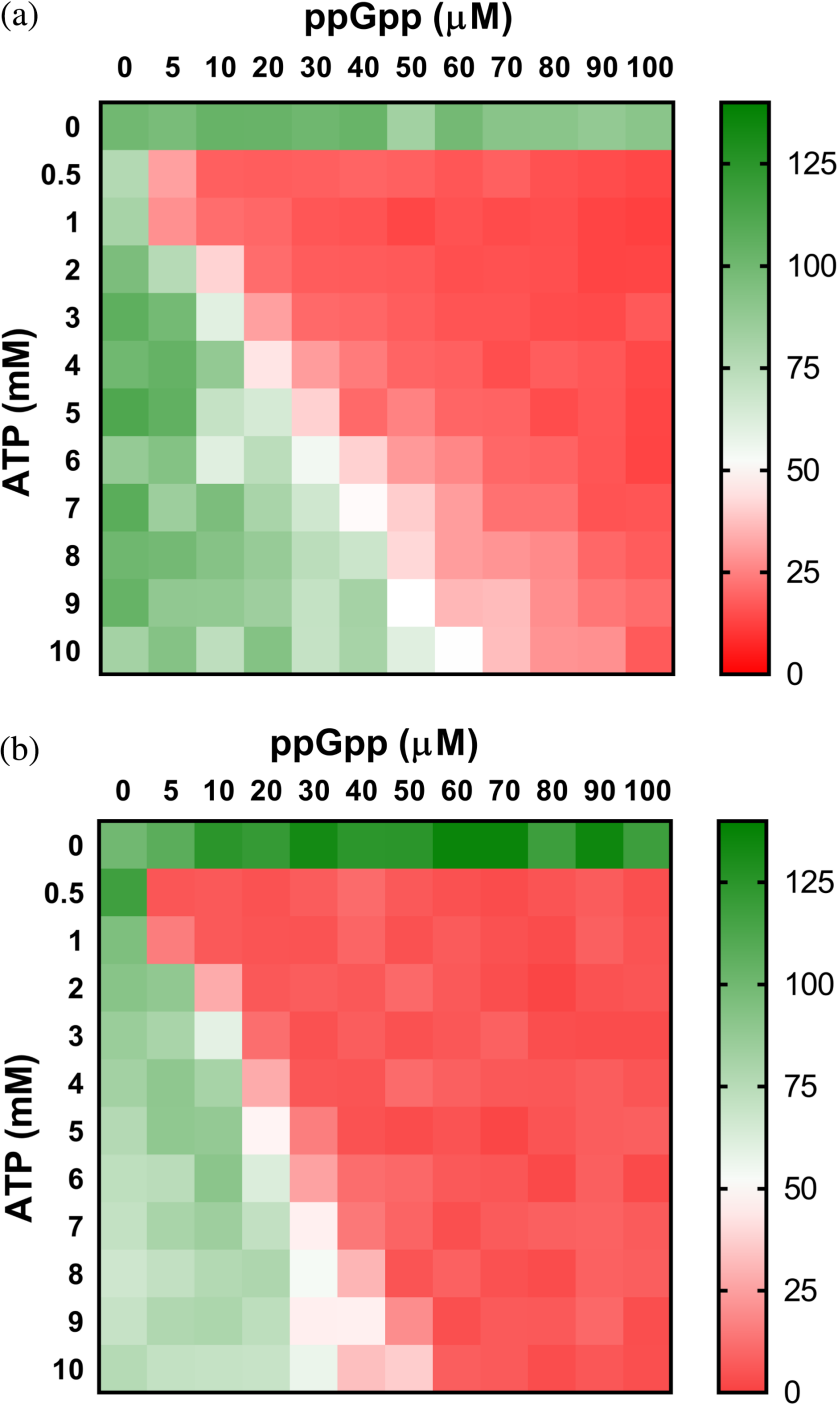
*ppGpp modulates the activity of IMPDH in Actinobacteria and Firmicutes*. Heatmap representation of the enzymatic percent activity (*V*_0_ values normalized to the *V*_0_ values in the absence of nucleotide) of BsIMPDH (a) and StcIMPDH (b) at different ATP versus ppGpp concentrations. The *V*_0_ values used for normalization are BsIMPDH 32.9 and StcIMPDH 7.6 nM s^−1^. BsIMPDH, *Bacillus subtilis* IMPDH; IMPDH, IMP dehydrogenase; StcIMPDH, *Streptomyces coelicolor* IMPDH

The results reported above indicate that occupancy of either the canonical Site 2 by ATP or the (p)ppGpp binding pocket is mutually exclusive. We then tested the activity of IMPDH in vitro in the presence of different amounts of ATP and ppGpp, at IMP and NAD^+^ concentrations within the expected intracellular range.^18^, ^33^, ^36^ Figure 7 shows that, within the assayed ATP concentration range, ppGpp can strongly inhibit the enzyme activity even at basal concentrations, in the mid micromolar range.^41^

## 3 DISCUSSION

Purine nucleotides are essential metabolites, involved in multiple metabolic pathways and cellular functions that need a fine‐tuned balance between adenine and guanine derivatives. Maintenance of GTP levels across species is critical to fitness, and GTP dysregulation has relevance to malignancy, genetic disease and genomic instability.^46^, ^47^, ^48^ In some gram‐positive bacteria, including *B. subtilis*, the intracellular levels of GTP must be maintained within a narrow range and excess GTP is severely detrimental for cell growth and survival.^30^, ^31^, ^38^ On the other hand, high GTP levels do not lead to a loss of viability in Proteobacteria, such as *E. coli*, although they inhibit cell growth.^49^, ^50^ In any case, despite the notable differences in tolerance to excess purine nucleotide levels among bacteria, the tight control of the purine nucleotide biosynthetic pathways must be a key facet of the cell homeostasis and the global metabolic response to environmental and nutritional changes. It is evident, for instance, that bacteria need to tightly modulate the purine biosynthetic pathways in response to the availability of these nucleotides in the culture media or downregulate them in conditions of nutritional stress, as part of the stringent response.^49^ Thereby, it is essential to elucidate the mechanisms, most probably redundant, that regulate purine nucleotide biosynthesis and keep the ATP/GTP ratio within a narrow physiological range within the cell.

In eukaryotic cells, IMPDH allosteric regulation, mediated by the binding of adenine and guanine nucleotides to the regulatory Bateman domain, plays an essential role in the maintenance of the balance between adenine and guanine nucleotide pools and GTP homeostasis. The physiological relevance of this mechanism of regulation is stressed by the fact that missense mutations that map in the allosteric binding sites of human IMPDHs are associated to severe retinopathies and dystonia.^20^, ^22^

In bacterial cells, however, no physiological mechanism of allosteric inhibition of IMPDH has been reported. In this study, we demonstrate that (p)ppGpp is a potent allosteric inhibitor of the IMPDHs from most bacterial phyla, except Proteobacterias, whose IMPDHs are allosterically controlled by the intracellular ratio of ATP/GTP. We found that a, previously ignored, key point to unveil bacterial IMPDH allosteric inhibition in vitro is the requirement of simultaneous binding of adenine and guanine nucleotides to the allosteric sites. Different from the eukaryotic enzymes, bacterial IMPDHs need ATP bound to the canonical Site 1 to be inhibited by guanine nucleotides that bind to either the canonical Site 2 (GTP/GDP) or a contiguous pocket ((p)ppGpp). This is most possibly the primary reason why guanine nucleotides have been previously unnoticed as allosteric inhibitors of bacterial IMPDHs in vitro.^7^, ^10^, ^30^, ^32^ Indeed, from the analysis of the high‐resolution structures of eukaryotic and prokaryotic enzymes, it is hard to define the structural determinants responsible for the different nucleotide specificities of the canonical allosteric sites in the Bateman domain of IMPDHs.

Given that the intracellular concentrations of ATP are millimolar^18^, ^33^, ^36^ and the ATP affinities for bacterial IMPDHs are in the micromolar range,^6^, ^10^, ^51^ it is expected that purine nucleotide‐induced octamers are the most abundant species in the cytoplasm. A recent report indicates that this might also be the case for eukaryotic IMPDHs, as crystals of recombinant *Trypanosoma brucei* IMPDH grown in the cytoplasm of intact insect cells show octamers that contain ATP bound to the canonical Site 1 of the Bateman domain.^15^ With ATP bound to the canonical Site 1, the activity of bacterial IMPDHs is then modulated by the mutually exclusive binding of adenine nucleotides to the canonical Site 2 and guanine nucleotides to either this site or the newly discovered (p)ppGpp binding site, which partially overlaps with the former. The occupancy of these sites determines the conformation of the enzyme: high ATP/GTP or ATP/(p)ppGpp ratios favor the extended conformation of catalytically active octamers. In contrast, when these ratios drop, octamer compaction occurs to inhibit the enzymatic activity.

We have recently reported that phosphorylation of residues in the nucleotide‐binding sites of the Bateman domain modulates the allosteric regulation of the retinal isoforms of human IMPDH.^23^ By analogy, it might then be plausible that post‐translational modifications control the allosteric regulation of bacterial IMPDHs. According to public databases (PLMD^52^ and dbPSP^53^), lysine acetylation is a recurrent modification within the Bateman domain of bacterial IMPDH. Within this domain, acetylation of residue K203 in *E. coli*, and the equivalent K206 in *B. subtilis*, called our attention because is evolutionarily conserved (Figure S8) and directly involved in the binding of GTP (Figure 3) and ppGpp (Figure 6). As an initial approach, we tested the effects of acetylation in vitro by using the K‐to‐Q point mutation that has been previously described to simulate the acetylation‐dependent neutralization of the positive lysine charge.^54^ Figure S11 shows how K203Q and K206Q substitutions significantly compromise allosteric inhibition in *E. coli* and *B. subtilis* enzymes, respectively. Although not conclusive, these results definitively encourage to perform new experiments to decipher if the allosteric regulation of bacterial IMPDHs is fine‐tuned by post‐translational modifications.

We propose the Bateman domain of IMPDH as a new crosstalk point between the adenine and guanine nucleotide pathways downstream IMP. This regulatory point, together with the crosswise utilization of GTP and ATP as cosubstrates of Adenylosuccinate and GMP synthases, respectively, might help to adjust the balance of purine nucleotides according to the cell metabolic demands. Our in vitro data on the allosteric inhibition of IMPDH is in good agreement with previously published in vivo data, since the deletion of the Bateman domain of IMPDH in *E. coli*, results in altered purine nucleotide concentrations and the inability to maintain the ATP/GTP balance within a fairly narrow physiological range.^18^, ^29^ Nonetheless, although our data points to a key role of the allosteric modulation of IMPDH by the intracellular ratios of ATP/GTP, we cannot discard additional mechanisms that contribute to maintain the purine nucleotide balance and GTP homeostasis. These mechanisms might imply the putative moonlighting functions of IMPDH,^29^, ^55^, ^56^, ^57^ as well as different enzymes.

The alarmones (p)ppGpp bind to a conserved ‐previously unrecognized‐ high affinity (p)ppGpp binding pocket within the Bateman domain that partially overlaps with the canonical Site 2. Multiple sequence alignments of bacterial IMPDH and phylogenetic analysis show that this site is present in most bacterial phyla, except for some classes of Proteobacteria, including γ and the closely related α‐ and ß‐proteobacteria, as well as some δ‐Proteobacteria genera. In contrast, ε‐Proteobacteria retained the (p)ppGpp binding site (Figures S8–S10) and the corresponding allosteric inhibition, as we have experimentally demonstrated for *Helicobacter pylori* IMPDH (HpIMPDH; Figure S12). The IMPDH phylogenetic tree, together with the species tree, allows us to propose that the bacterial IMPDH ancestor contained the (p)ppGpp binding site, and this was lost during the evolution of the proteobacterial lineage.

Remarkably, a similar evolutionary history has been reported for the enzymes guanylate kinase (Gmk, downstream from IMPDH in the guanine nucleotide de novo pathway^58^) and hypoxanthine phosphoribosyltransferase (HprT, GPRT in Figure 1, in the *salvage* pathway^59^). (p)ppGpp inhibit bacterial IMPDHs with IC_50_ in the low microM range, similar to the IC_50_ values reported for Gmk and HprT,^58^, ^59^ which represent basal levels of these alarmones.^41^ Therefore, our results confirm the vital housekeeping function of (p)ppGpp on GTP homeostasis. They tightly control the guanine nucleotide de novo and *salvage* pathways in response to both extrinsic stress and intrinsic cell status, buffering GTP against fluctuations and preserving metabolic stability.

The loss of (p)ppGpp inhibition of the enzymes IMPDH, Gmk, and HprT during the evolution of the proteobacterial lineage was paralleled with the acquisition of (p)ppGpp regulation by the RNA polymerase (RNAP). This is demonstrated by the presence of the MAR motif at the N‐terminal region of the ω‐subunit of RNAP in α, β, δ, and γ, but neither in ε‐proteobacteria nor in most other bacterial phyla^60^ (Figure S10). Moreover, it is plausible to propose that the loss of the tight control exerted by basal levels of (p)ppGpp over IMPDH, Gmk, and HprT enzymes is possibly correlated with the low GTP toxicity in Proteobacteria.^50^ In any case, these observations represent fascinating examples on how evolution has found different (p)ppGpp targets and rewired regulatory networks to achieve the same regulatory ends.

Altogether, the results presented here indicate an essential role of IMPDH allosteric regulation on bacterial GTP homeostasis and further expand our knowledge about the crosstalk between (p)ppGpp signaling and the guanine nucleotide biosynthetic pathway. We demonstrate that the activity of bacterial IMPDHs is allosterically controlled by an evolutionarily conserved nucleotide‐controlled conformational switch that has been divergently adapted to the specific particularities of each organism. Moreover, we have identified significant differences in the mechanisms of regulation between eukaryotic and prokaryotic IMPDH enzymes, opening the door to the development of approaches to antibiotic discovery.

## 4 METHODS

### 4.1 Cloning, site‐directed mutagenesis, and protein purification

Open reading frames of the different enzymes were amplified by PCR using genomic DNA as template and inserted into an ad hoc modified pET15b bacterial expression vector with the thrombin cleavage site substituted by the tobacco echt virus protease recognition sequence. Site‐directed mutagenesis was performed using the QuikChange II method (Agilent Technologies). All plasmids were corroborated by DNA sequencing.

IMPDH enzymes were overexpressed overnight in *E. coli* BL21 (DE3) strain in terrific broth^61^ at 18°C and purified by immobilized metal affinity chromatography according to standard protocols. The 8‐histidine tail present at the N‐terminal of the overexpressed proteins was cleaved by overnight digestion at room temperature with tobacco etch virus protease. The cleaved proteins were then injected into a HiPrep Sephacryl S‐300 16/60 HR size‐exclusion chromatography column (Cytiva) equilibrated in buffer 20 mM Tris–HCl, 5% glycerol, 500 mM KCl, 1 mM DTT, pH 8.0. Fractions containing IMPDH proteins were pooled, concentrated at 4°C using a 10 kDa cutoff Amicon Ultra centrifugal filter (Millipore), aliquoted and stored at −80°C. All the enzymes showed at least 98% purity by SDS‐PAGE densitometric analysis and did not significantly lose activity after one cycle of freezing/thawing. Protein and nucleotide concentrations were determined spectrophotometrically.

### 4.2 Enzyme kinetics assays

IMPDH activity was assayed using 384‐well microtiter plates by monitoring the appearance of NADH by fluorescence (*λ*_exc_ = 340 nm and *λ*_em_ = 460 nm, using a 10 nm slit window for both excitation and emission).

The buffer used for the guanine nucleotide titration curves shown in Figures 2 and 5 were 100 mM Tris–HCl pH 8.0, 100 mM KCl, 2 mM MgCl_2_ (free), 2 mM DTT, 0.5 mM NAD^+^, 0.5 mM IMP, 0‐ or 0.25‐mM ATP, and 50 nM enzyme, measured at 28°C (BsIMPDH and StcIMPDH) or 32°C. The total amount of MgCl_2_ was adjusted for each nucleotide concentration to keep 1 mM free Mg^2+^ constant concentration, as previously described.^6^ The experimental data were fitted to the Michaelis–Menten and allosteric sigmoidal equations using GraphPad Prism (GraphPad Software).

The buffer used for the heat map plot shown in Figure 4 and 7 in the main text and Supplemental Figure 11 was 100 mM Tris‐HCl, pH 8.0, 100 mM KCl, 1 mM MgCl_2_, 2 mM DTT, 1 mM NAD^+^, 0.2 mM IMP. Nucleotides: ATP‐Mg^2+^, GTP‐Mg^2+^, and ppGpp‐Mg^2+^ were added at the indicated concentrations, and 20 nM of EcIMPDH, EcIMPDH‐K203Q, PaIMPDH, and StcIMPDH and 40 nM of BsIMPDH and BsIMPDH‐K206Q enzymes were used. Measurements we performed at 32°C for EcIMPDH and PaIMPDH and 28°C for BsIMPDH and StcIMPDH.

### 4.3 Protein crystallization and structure solution

Crystals of PaIMPDH‐ATP‐GDP were grown at 22°C in sitting drops using the vapor diffusion method by mixing a protein solution at 10 mg ml^−1^ in 5 mM Tris–HCl, 100 mM KCl, 0.5 mM ATP, 5 mM GDP, 3.52 mM total MgCl_2_ (1 mM free Mg^2+^ estimated as described in Reference 6), pH 8.0, with an equal volume of mother liquor corresponding to the condition D11 of the commercial screening Morpheus^62^: 0.02 M sodium formate; 0.02 M ammonium acetate; 0.02 M sodium citrate tribasic dihydrate; 0.02 M potassium sodium tartrate tetrahydrate; 0.02 M sodium oxamate, 12.5% v/v MPD; 12.5% PEG 1000; 12.5% w/v PEG 3350 in 0.1 M of the buffer system Tris (base), bicine, pH 8.5.

Crystals of StcIMPDH‐ATP‐ppGpp were obtained as before by mixing 10 mg ml^−1^ of StcIMPDH in buffer in 5 mM Tris–HCl, 100 mM KCl, 1.5 mM ATP, 0.5 mM ppGpp, 3 mM total MgCl_2_, pH 8.0 with equal volume of mother liquor corresponding to the condition H9 of the commercial screening Morpheus‐II^63^: 0.01 M spermine tetrahydrochloride, 0.01 M spermidine trihydrochloride, 0.01 M 1,4‐diaminobutane dihydrochloride, 0.01 M dl‐ornithine monohydrochloride, 15% w/v PEG 3000, 20% v/v 1,2,4‐butanetriol, 1% w/v NDSB 256, and 0.1 M of the buffer system Gly‐Gly, AMPD, pH 8.5.

Protein crystals were flashed‐cooled in liquid nitrogen and data were collected at 100 K, using monochromatic X‐rays of 1.00 Å wavelength, at the Diamond and ALBA synchrotrons. Diffraction intensities were indexed, integrated and anisotropically truncated by using the software autoPROC.^64^, ^65^ The structures were solved by molecular replacement with the program PHASER^66^ from the CCP4 software suite,^67^ using as template the structure of *P. aeruginosa* IMPDH (PDB ID 4AVF).^68^ The structural models were iteratively improved by alternating automated refinement, using the PHENIX crystallographic software package^69^ with manual modeling, using the program COOT.^70^ Simulated annealing (torsion coordinates), gradient‐driven positional, restrained individual isotropic B‐factor and TLS refinement^71^ were used for refinement. The figures showing three‐dimensional protein structures were generated using PyMOL.^72^

### 4.4 Small angle X‐ray scattering

SAXS measurements were performed at the B21 beamline in the Diamond synchrotron, using buffer: 20 mM Tris–HCl, 300 mM KCl, 3 mM DTT, 5% glycerol, pH 8.0 and a protein concentration of 2.5 mg ml^−1^ (PaIMPDH) and 3 mg ml^−1^ (StcIMPDH). Nucleotide concentrations were 2 mM ATP, 0.25 mM ATP + 1.5 mM GDP, or 1 mM ATP + 0.1 mM ppGpp. The total amount of MgCl_2_ was adjusted for each nucleotide concentration to keep 1 mM free Mg^2+^ constant concentration, as previously described.^6^ During the measurements, the beamline was used in the default configuration: a beam energy of 13 keV, a sample‐to‐detector distance of 3.7 m.^73^ The samples were flowing through an in‐vacuum cell, kept at 10°C, to minimize radiation damage.

All nondamaged protein frames were averaged and buffer scattering was subtracted using the ATSAS software suite.^74^ The theoretical scattering curves in Figure S4a were calculated from the PaIMPDH‐ATP (PDB ID 4DQW),^10^ PaIMPDH‐ATP‐GDP and PaIMPDH‐APO (PDB ID 6GJV)^51^ crystal structures using the program CRYSOL.^75^ The theoretical scattering curves in Figure S4b were calculated from the PaIMPDH‐ATP (PDB ID 4DQW),^10^ StcIMPDH‐ATP‐ppGpp (this work) and an isolated tetramer of PaIMPDH‐ATP (for StcIMPDH‐APO).

### 4.5 Molecular dynamics simulations

Previous to the TMD procedures, crystal structures were subjected to 100 ns of unrestrained molecular dynamics (MD) simulations in presence of the different ligands using the AMBER18 MD package (http://ambermd.org; University of California‐San Francisco), essentially as previously described.^6^, ^76^ The structures were solvated with a periodic octahedral pre‐equilibrated solvent box using the LeaP module of AMBER, with 12 Å as the shortest distance between any atom in the protein subdomain and the periodic box boundaries. MD simulation was performed using the PMEMD program of AMBER18 and the ff14SB force field (http://ambermd.org), applying the SHAKE algorithm, a time step of 2 fs and a nonbonded cutoff of 12 Å. Systems were initially relaxed over 10,000 steps of energy minimization, using 1000 steps of steepest descent minimization followed by 9,000 steps of conjugate‐gradient minimization. Simulations were then started with a 20 ps heating phase, raising the temperature from 0 to 300 K in 10 temperature change steps, after each of which velocities were reassigned. During minimization and heating, the Cα trace dihedrals were restrained with a force constant of 500 kcal mol^−1^ rad^−2^ and gradually released in an equilibration phase in which the force constant was progressively reduced to 0 over 200 ps. After the equilibration phase, 100 ns of unrestricted MD simulation were obtained for the structures

To compare the work and force required to adopt the active (extended) conformation from the inhibited (compacted) conformation, and vice versa, of both PaIMPDH and StcIMPDH monomers in the presence of the different ligands, the calculation of the accumulated work (kcal mol^−1^) and force (kcal mol^−1^ Å^−1^) was performed for each case using TMD. In all cases, a spring constant of 5 kcal mol^−1^ Å^−2^ was used and the whole trajectory was divided into 1,000 discrete steps of 0.1 ns per step and a final root main square deviation (rmsd) of 2.0 Å. For each calculation step, rmsd values were recorded to later reconstruct the forces and works generated along with each trajectory. MD and TMD trajectories were analyzed using VMD software.^77^

### 4.6 Multiple sequence alignment and phylogenetic analysis

IMPDH protein sequences were obtained by recursive BLAST^78^ searches at NCBI and aligned with Mafft v7.305b.^79^ Alignments were inspected with Jalview^80^ and cleaned for unreliably aligned regions using Trimal v1.4.rev5,^81^ removing sites containing gaps in more than 50% of the sequences (−gt 0.5). Phylogenetic trees were reconstructed with Phyml 3.0.^82^ Best fit models of evolution were identified with SMS,^83^ and 100 bootstrap replicates were requested. Phylogenetic trees were edited with FigTree (http://tree.bio.ed.ac.uk/software/figtree).

## AUTHOR CONTRIBUTIONS

**David Fernández‐Justel:** Investigation (equal). **Íñigo Marcos‐Alcalde:** Investigation (equal). **Federico Abascal:** Investigation (equal). **Nerea Vidaña:** Investigation (equal). **Paulino Gómez‐Puertas:** Investigation (equal). **Alberto Jiménez:** Investigation (equal). **José L. Revuelta:** Conceptualization (equal); funding acquisition (supporting); project administration (equal). **Rubén M. Buey:** Conceptualization (lead); funding acquisition (lead); investigation (lead); project administration (equal); writing – original draft (lead); writing – review and editing (lead).

## Supporting information


**Table S1.** X‐ray crystallography and data collection and refinement statistics.
**Figure S1.** Effects of GDP (A) and pppGpp (B) on the catalytic activity of IMPDH in vitro.
**Figure S2.** The compaction of octamers mediates the allosteric inhibition of PaIMPDH.
**Figure S3.** Mutational analysis of the allosteric binding sites.
**Figure S4.** The conformational switch of IMPDH observed in solution by SAXS.
**Figure S5.** GTP/GDP and (p)ppGpp binding to the Bateman domain strongly stabilize the inhibited IMPDH conformation.
**Figure S6.** Allosteric inhibition of GTP at physiological ATP concentration.
**Figure S7.** Eukaryotic and bacterial IMPDHs adopt similar compact conformation upon allosteric inhibition.
**Figure S8.** Sequence alignment of the Bateman domain of selected prokaryotic IMPDHs.
**Figure S9.** Evolutionary analysis of bacterial IMPDHs.
**Figure S10.** Tree of life of selected organisms.
**Figure S11.** Lysine acetylation fine‐tune bacterial IMPDH allosteric regulation in vitro.
**Figure S12.** Effects of guanine nucleotides on the catalytic activity of HpIMPDH in vitro.

**Video S1** Supplementary Video.

**Video S2** Supplementary Video.
