# Supplementary material for: Diversity of mechanisms to control bacterial GTP homeostasis by the mutually exclusive binding of adenine and guanine nucleotides to IMP dehydrogenase
Source: Protein Sci. 2022 Apr 27;31(5):e4314. doi: 10.1002/pro.4314 (PMC9462843; doi:10.1002/pro.4314)
Supplement: Supplementary file 1 — Table S1. X‐ray crystallography and data collection and refinement statistics. Figure S1. Effects of GDP (A) and pppGpp (B) on the catalytic activity of IMPDH in vitro. Figure S2. The compaction of octamers mediates the allosteric inhibition of PaIMPDH. Figure S3. Mutational analysis of the allosteric binding sites. Figure S4. The conformational switch of IMPDH observed in solution by SAXS. Figure S5. GTP/GDP and (p)ppGpp binding to the Bateman domain strongly stabilize the inhibited IMPDH conformation. Figure S6. Allosteric inhibition of GTP at physiological ATP concentration. Figure S7. Eukaryotic and bacterial IMPDHs adopt similar compact conformation upon allosteric inhibition. Figure S8. Sequence alignment of the Bateman domain of selected prokaryotic IMPDHs. Figure S9. Evolutionary analysis of bacterial IMPDHs. Figure S10. Tree of life of selected organisms. Figure S11. Lysine acetylation fine‐tune bacterial IMPDH allosteric regulation in vitro. Figure S12. Effects of guanine nucleotides on the catalytic activity of HpIMPDH in vitro. [file PRO-31-e4314-s001.pdf]

SUPPLEMENTAL INFORMATION

Diversity of mechanisms to control bacterial GTP homeostasis by the mutually exclusive binding of adenine and guanine nucleotides to IMP dehydrogenase

David Fernández-Justel[1], Íñigo Marcos-Alcalde[2,3], Federico Abascal[4], Nerea Vidaña[1], Paulino Gómez-Puertas[2], Alberto Jiménez[1], José L Revuelta[1] and Rubén M Buey[1*]

[1]Metabolic Engineering Group. Dept. Microbiology and Genetics. Universidad de Salamanca

[2] Molecular Modeling Group, Centro de Biología Molecular Severo Ochoa, CBMSO (CSIC-UAM), 28049 Madrid, Spain

[3] Biosciences Research Institute, School of Experimental Sciences, Universidad Francisco de Vitoria, 28223. Pozuelo de Alarcón, Madrid, Spain

[4] Wellcome Sanger Institute. Hinxton, UK

[*]To whom correspondence should be addressed: ruben.martinez@usal.es

Supplemental Table 1. *X-ray crystallography and data collection and refinement statistics.*

	PaIMPDH ATP-GDP	StcIMPDH ATP-ppGpp
**PDB ID**	**7PJI**	**7PMZ**
**Wavelength (Å)**	1.00	1.00
**Resolution (Å)**	1.65	2.03
**Space group**	I4	$P2_1$
**Unit cell (Å / º)**	120.95 120.95 145.49<br>90.00 90.00 90.00	119.29 300.90 123.09<br>90.00 90.29 90.00
**Unique reflections**	84861 (4228)	474239 (23712)
**Multiplicity**	14.0 (13.9)	4.6 (4.5)
**Completeness spherical (%)**	67.8 (17.8)	84.9 (27.1)
**Completeness ellipsoidal (%)**	95.7 (69.7)	94.2 (58.1)
**Mean I/sigma(I)**	16.8 (1.5)	9.4 (1.5)
**Wilson B-factor**	25.18	30.62
**R-merge**	0.09 (1.76)	0.10 (0.99)
**R-meas**	0.09 (1.83)	0.11 (1.13)
**R-pim**	0.03 (0.49)	0.055 (0.53)
**CC1/2**	0.999 (0.620)	0.998 (0.589)
**Reflections used in refinement**	84628 (880)	474175 (9428)
**Reflections used for R-free**	4286 (44)	23521 (454)
**R-work**	0.18 (0.32)	0.18 (0.26)
**R-free**	0.20 (0.33)	0.21 (0.30)
**Non-H atoms:**	7258	53302
**macromolecules**	6476	48630
**ligands**	182	1456
**solvent**	654	3569
**Protein residues**	892	6842
**RMS(bonds)**	0.007	0.009
**RMS(angles)**	1.06	1.19
**Ramachandran favored (%)**	98.06	97.83
**Ramachandran allowed (%)**	1.94	2.13
**Ramachandran outliers (%)**	0.00	1.0
**Rotamer outliers (%)**	0.16	0.93
**Clashscore**	4.32	4.08
**Average B-factor:**	35.28	39.31
**macromolecules**	34.61	39.22
**ligands**	35.88	38.80
**solvent**	42.25	40.66

Statistics for the highest-resolution shell are shown in parentheses.

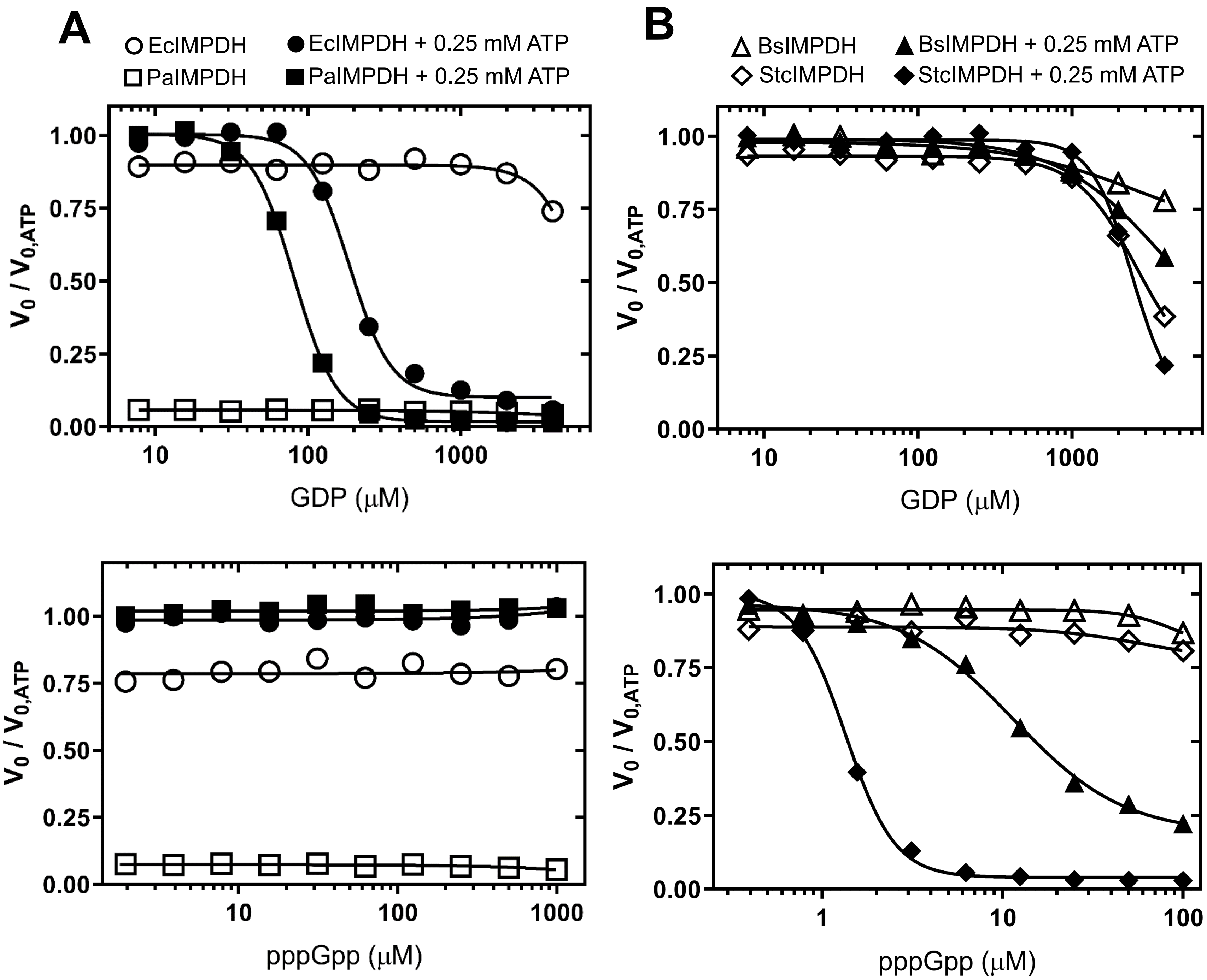

Supplemental Figure 1. *Effects of GDP (A) and pppGpp (B) on the catalytic activity of IMPDH in vitro*. Graphs showing the normalized initial velocity values (V_0 values in the absence of GDP or pppGpp divided by the respective values in the presence of GDP or ppGpp. The V_0 values used for the normalization of the data are: EcIMPDH 18.5 ± 1.0, PaIMPDH 26.7 ± 0.9, BsIMPDH 12.9 ± 0.8, and StcIMPDH 12.6 ± 0.4 nM/sec (mean ± std. error). Estimated IC_{50} values for GDP are 187.2 ± 8.8 μM μM and 81.3 ± 1.0 μM (mean ± std. error) for BsIMPDH and StcIMPDH, respectively. Estimated IC_{50} values for pppGpp are 11.4 ± 1.2 μM and 1.4 ± 0.03 μM (mean ± std. error) for BsIMPDH and StcIMPDH, respectively.

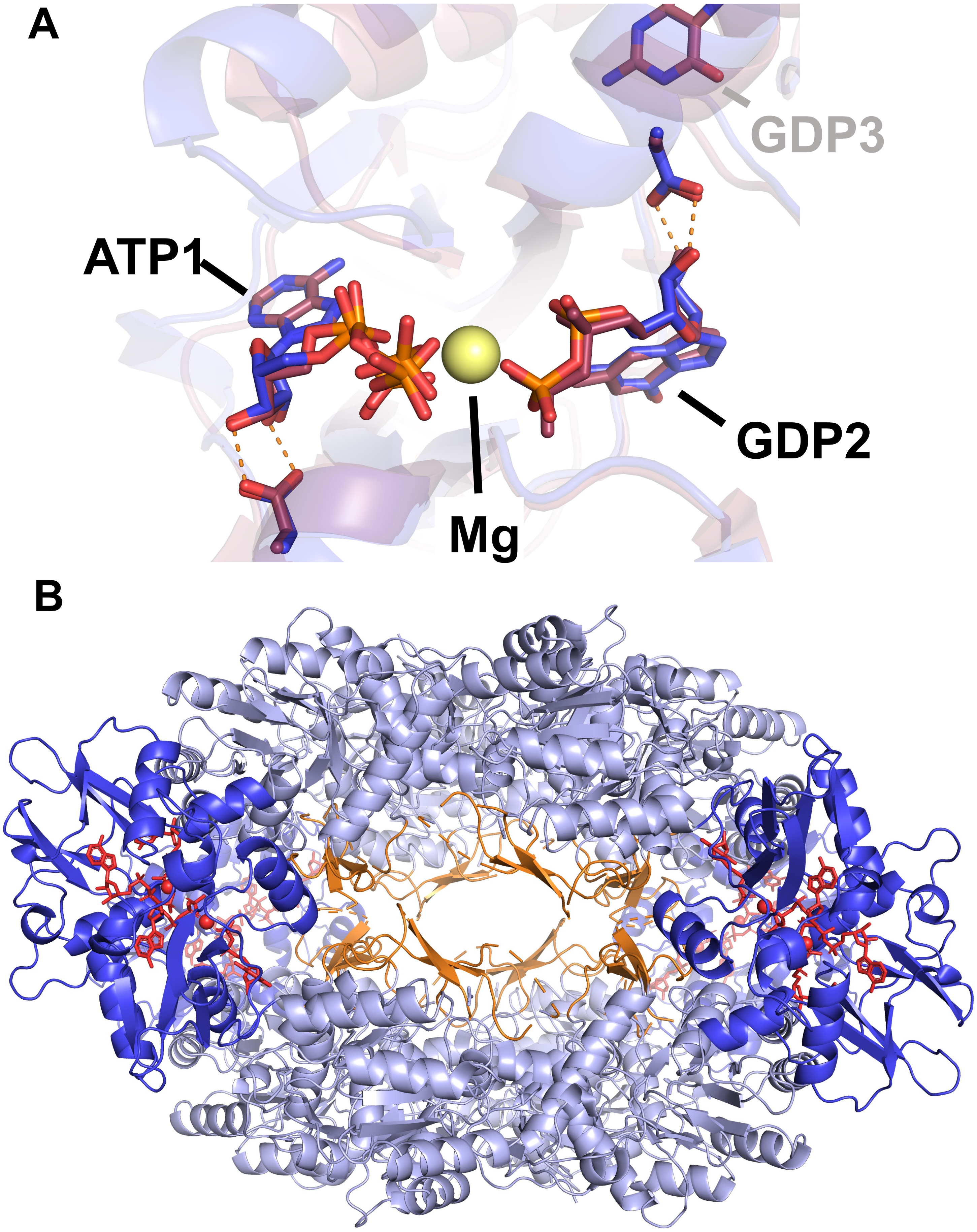

Supplemental Figure 2. *The compaction of octamers mediates the allosteric inhibition of PaIMPDH.* **A.** Structural superimposition of the structures of PaIMPDH-ATP/GDP (blue ribbons) and AgIMPDH-ATP/GDP/GDP (red ribbons; PDB ID 5TC3), showing the identical binding modes of the nucleotides on the canonical sites of the Bateman domain. **B.** Octameric structure of PaIMPDH-ATP/GDP reconstructed from the crystallographic lattice contacts, showing a compacted octamer where the finger domains (orange ribbons) of opposing tetramers are forced to interact. The catalytic and the regulatory Bateman domains are colored in light and dark blue, respectively. Bound nucleotides are shown in red sticks.

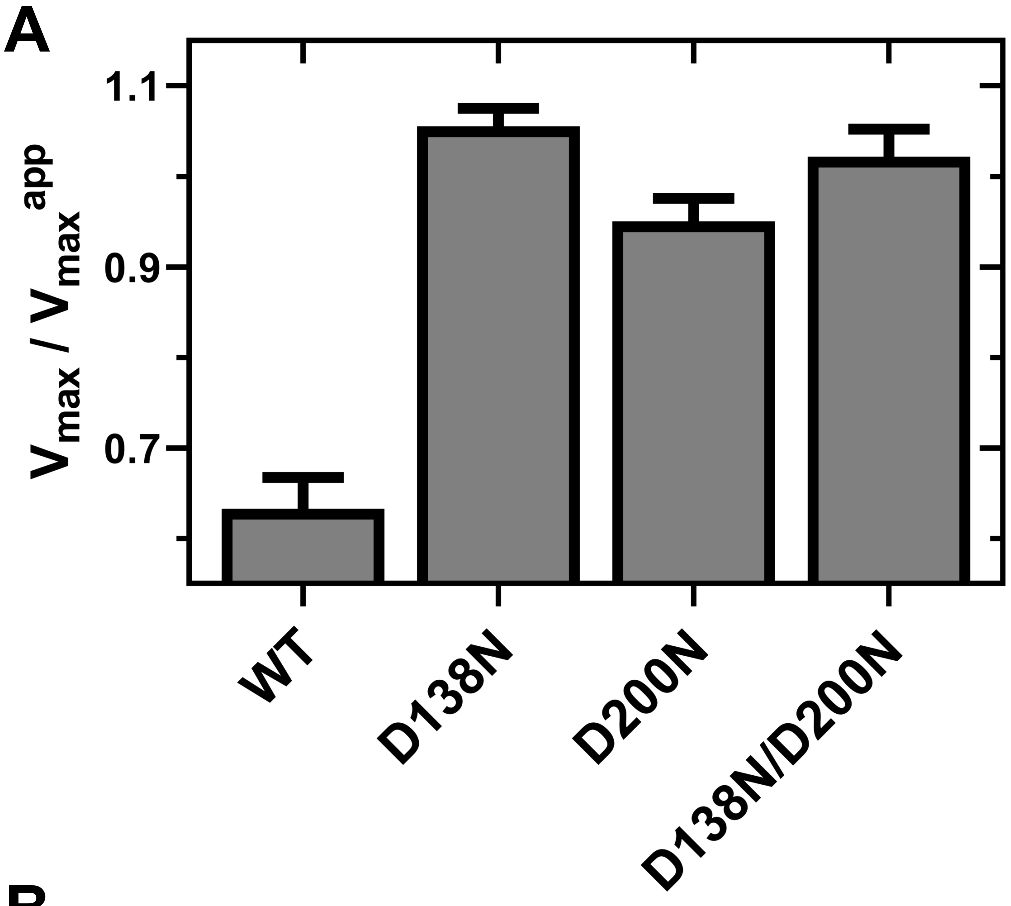

	Vmax (nM/sec)
**WT**	30.22 ± 0.93
**D138N**	22.82 ± 0.51
**D200N**	22.78 ± 0.51
**D138N/D200N**	14.79 ± 0.41

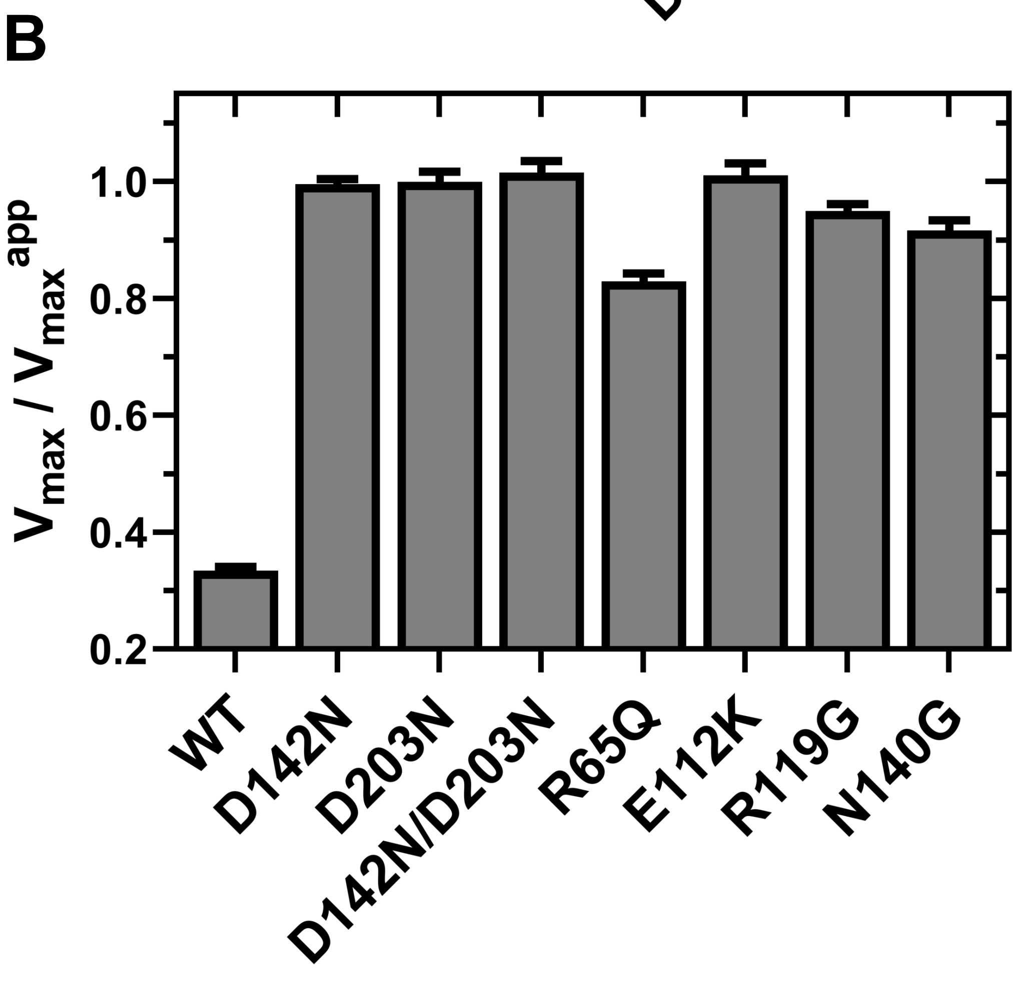

	Vmax (nM/sec)
**WT**	22.24 ± 0.37
**D142N**	18.05 ± 0.28
**D203N**	15.26 ± 0.31
**D142N/D203N**	16.37 ± 0.29
**R65Q**	17.24 ± 0.24
**E112K**	21.79 ± 0.42
**R119G**	20.19 ± 0.19
**N140G**	22.95 ± 0.24

Supplemental Figure 3. *Mutational analysis of the allosteric binding sites*. Normalized V_{max} values (V_{max} in the presence of ATP / V_{max} in the presence of ATP and guanine nucleotides; mean and standard deviations) for the WT and different mutants of EcIMPDH (**A**) and BsIMPDH (**B**). Nucleotide concentrations for EcIMPDH were: 0.25 mM ATP and 1 mM GTP, measured at 32ºC, and for BsIMPDH: 1 mM ATP and 50 µM ppGpp, measured at 28ºC. The enzymatic reactions were performed in all cases using 50 nM enzyme in bufffer 100 mM TrisHCl, pH 8.0, 100 mM KCl, 1 mM DTT, 1 mM free $MgCl_2$, 0.5 mM NAD and IMP concentrations ranging from 4 µM to 5mM. The V_{max} values used for normalization are shown in the tables.

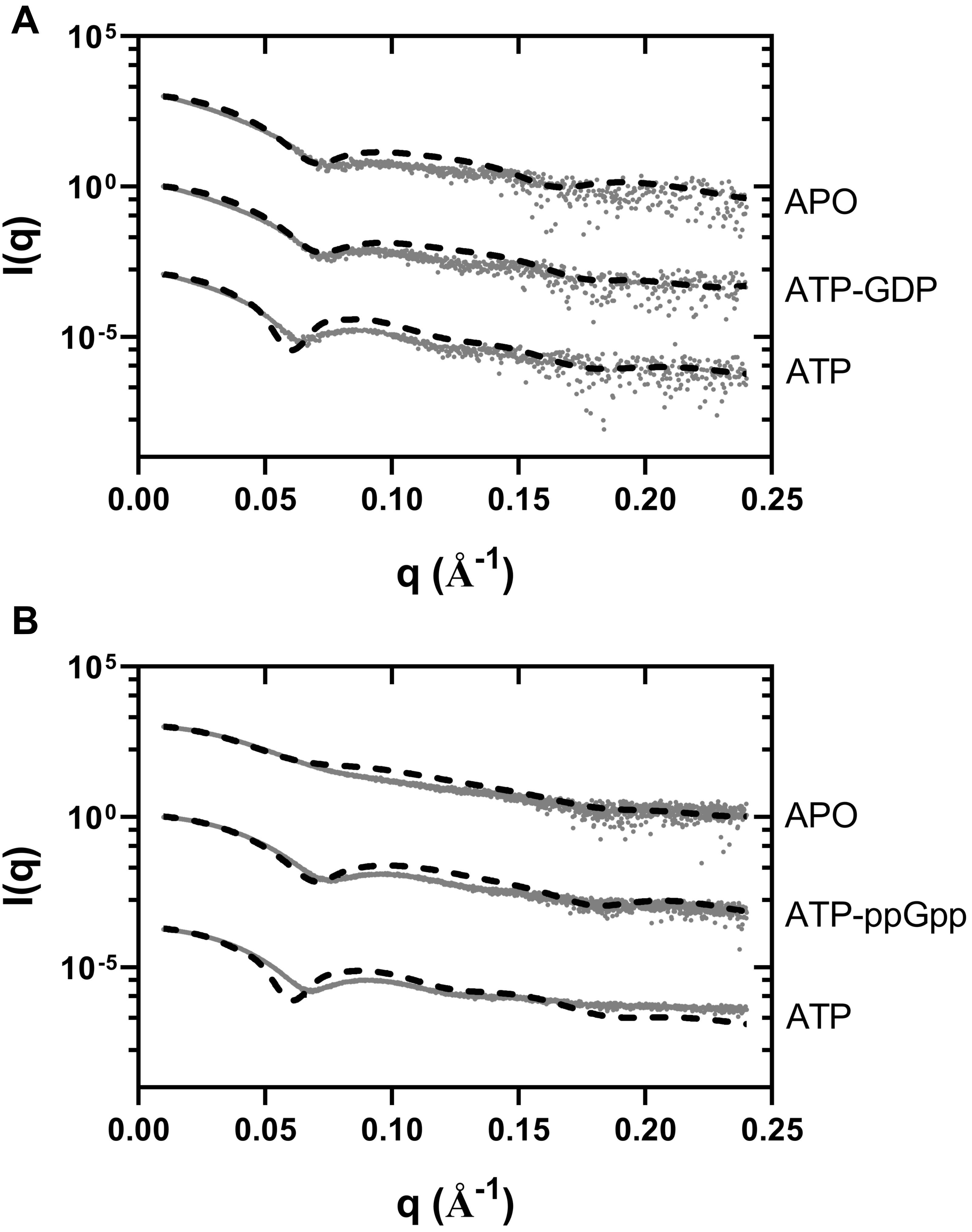

Supplemental Figure 4. *The conformational switch of IMPDH observed in solution by SAXS.* SAXS profiles of PaIMPDH (**A**) and StcIMPDH (**B**) in the presence of different nucleotides. To facilitate visualization, the curves have been conveniently displaced along the y axis. At the SAXS resolution, the profiles for ATP-GTP and ATP-pppGpp are indistinguishable from ATP-GDP and ATP-ppGpp, respectively.

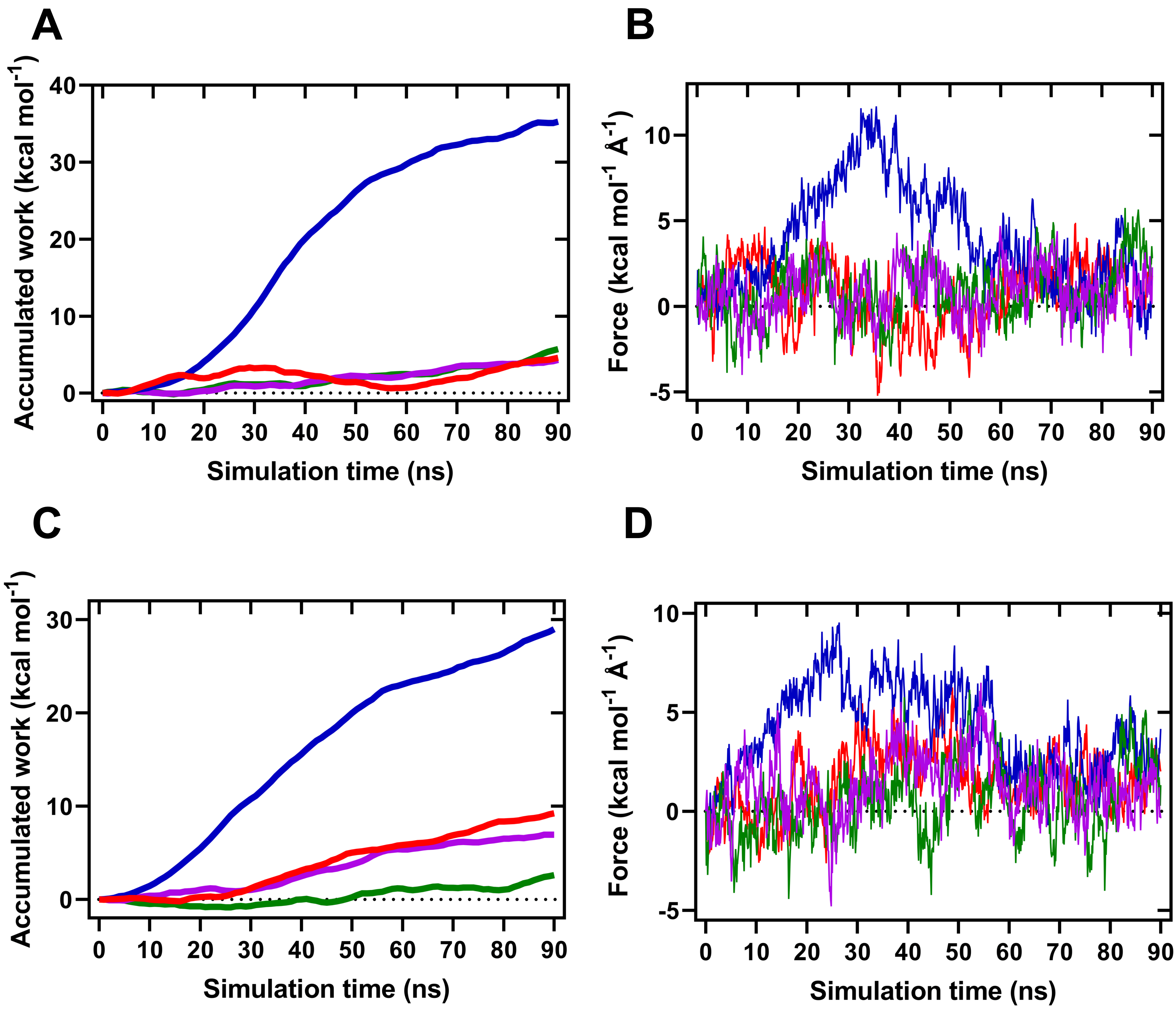

Supplemental Figure 5. ***GTP/GDP and (p)ppGpp binding to the Bateman domain strongly stabilize the inhibited IMPDH conformation.*** Accumulated work and forces exerted along the targeted molecular dynamics simulations of monomers of PaIMPDH (panels **A** and **B**) and StcIMPDH (panels **C** and **D**) induced to adopt a compact conformation, starting from the extended one, in the presence of ATP1/ATP2 (purple lines), ATP1/GDP2 or ATP1/ppGpp (green lines). Alternatively, IMPDH monomers were induced to adopt an extended conformation, starting from the compacted one, in the presence of ATP1/ATP2 (red lines), ATP1/GDP2 or ATP1/ppGpp (blue lines), monitoring also the ccumulated work and the forces applied during the process.

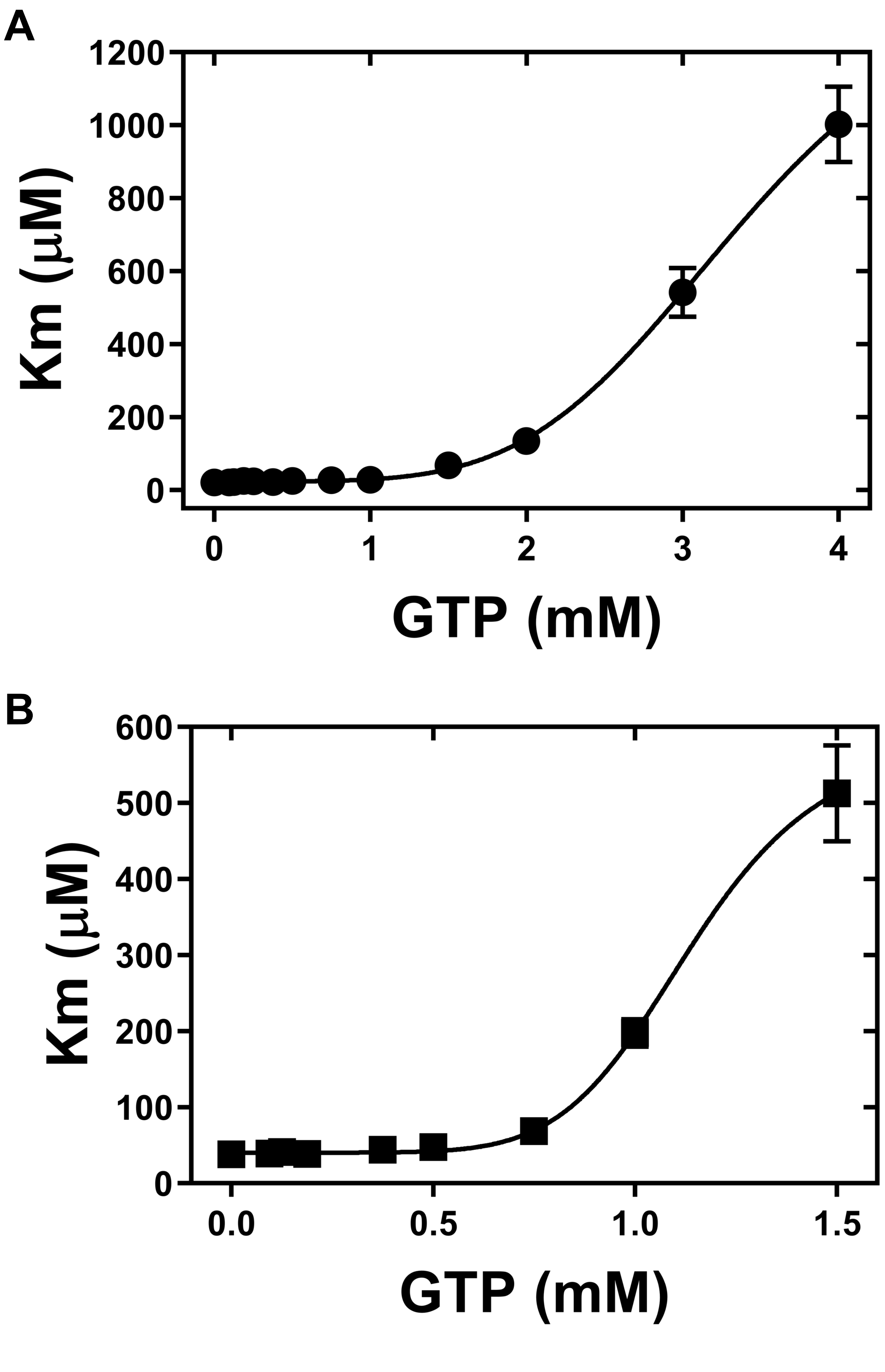

Supplemental Figure 6. *Allosteric inhibition of GTP at physiological ATP concentration.* GTP titration curves of EcIMPDH (**A)** and PaIMPDH (**B)** measured at 32ºC in buffer 100 mM Tris-HCl, pH 8.0, 100 mM KCl, 1 mM DTT, 1 mM free $MgCl_2$, 20 nM enzyme, 3 mM ATP, 0.5 mM NAD and IMP concentrations ranging from 4 µM to 5mM. The Km values in the absence of GTP were 23.45 ± 1.47 µM for EcIMPDH and 36.96 ± 1.62 µM for PaIMPDH.

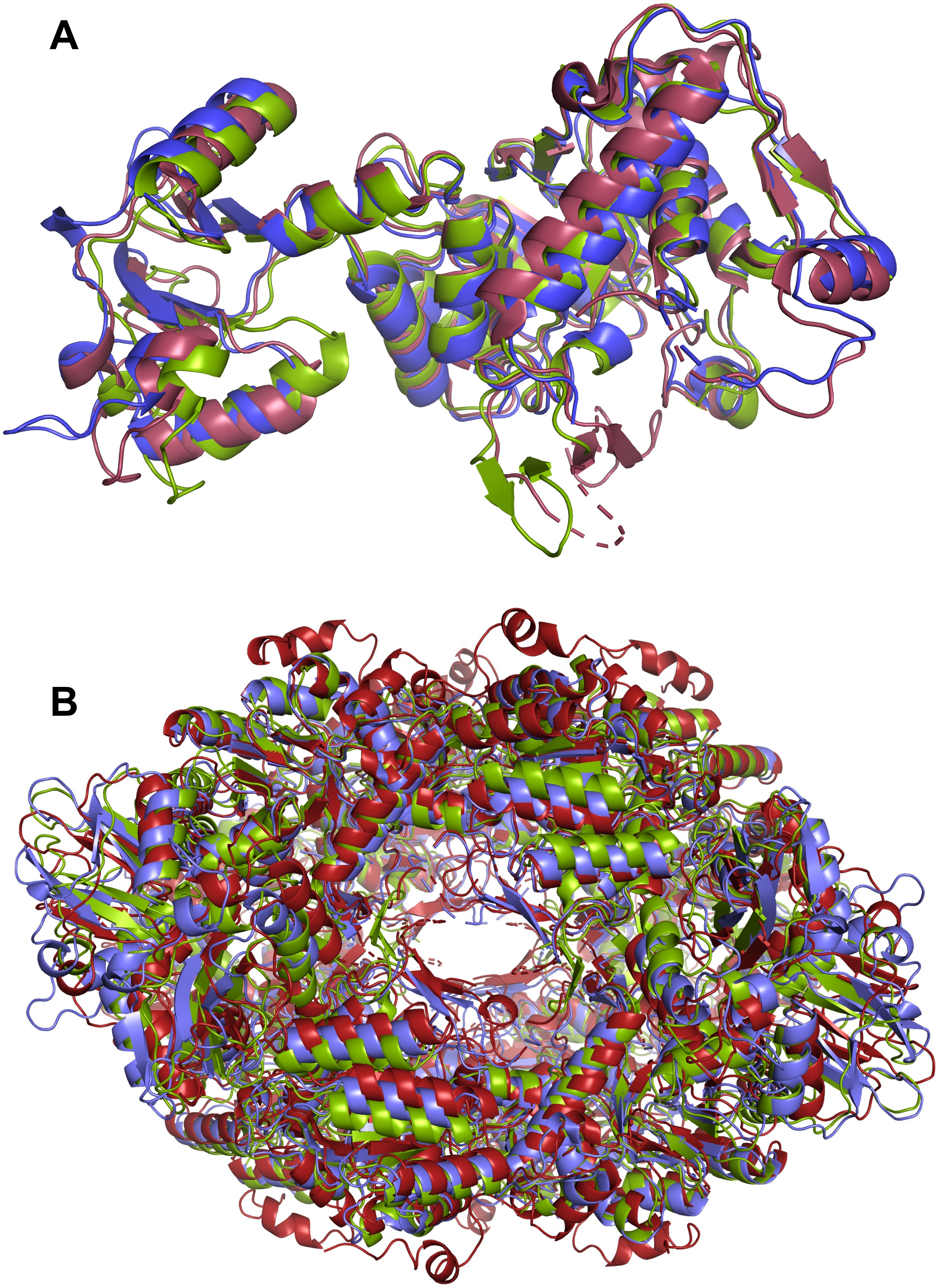

Supplemental Figure 7. *Eukaryotic and bacterial IMPDHs adopt similar compact conformation upon allosteric inhibition*. **A.** Structural comparison of the conformation adopted by IMPDH monomers, upon allosteric inhibition. Blue ribbons: PaIMPDH-ATP/GDP; Green ribbons: StcIMPDH-ATP/ppGpp; Red ribbons: AgIMPDH-ATP/GDP/GDP (PDB ID 5TC3). **B.** Structural superimposition of the octamers formed by the monomers in panel A.

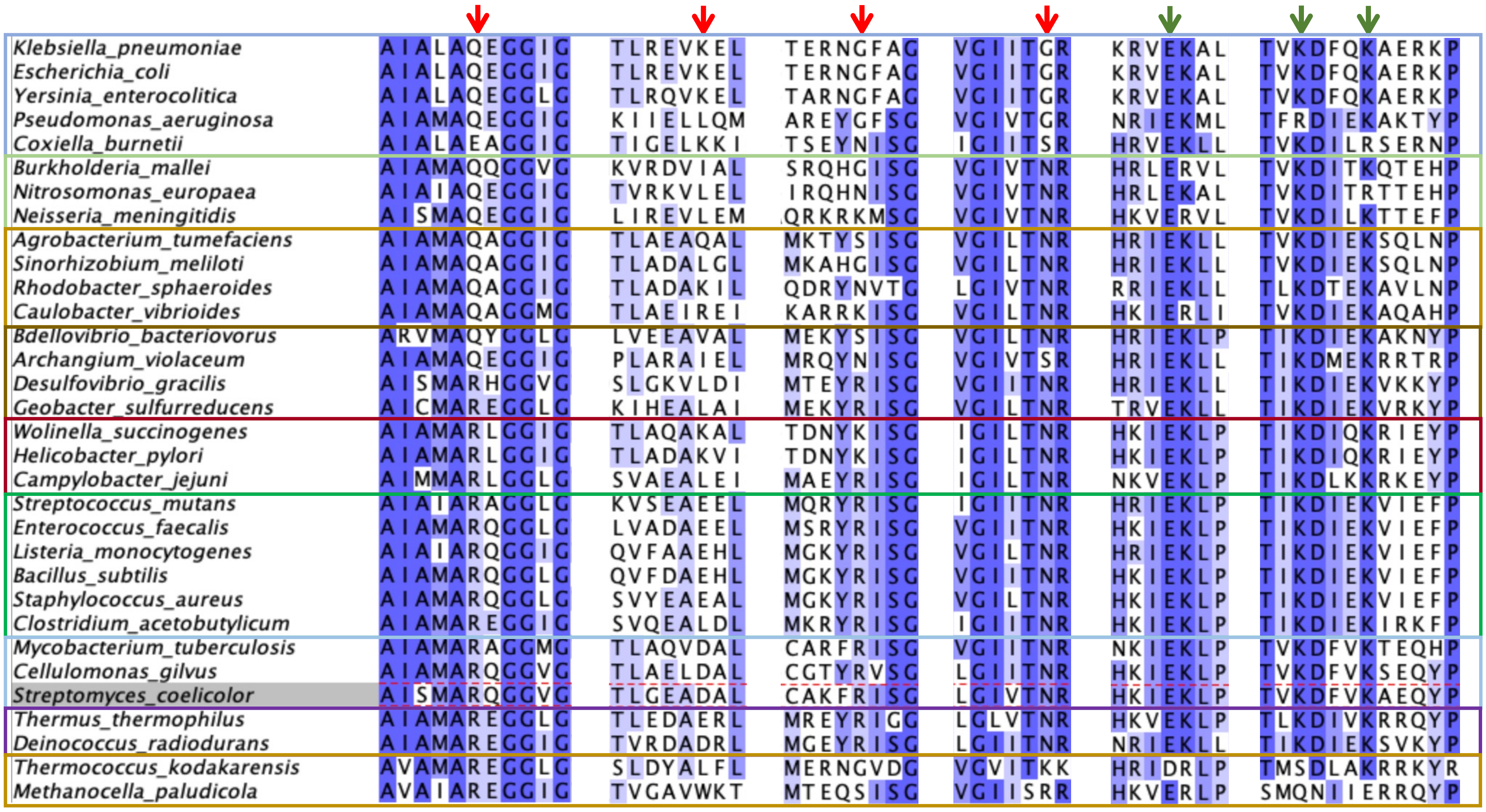

Supplemental Figure 8. *Sequence alignment of the Bateman domain of selected prokaryotic IMPDHs.* Fragment of a multiple sequence alignment of the Bateman domain of selected bacterial IMPDHs. The red arrows indicate key nucleotide-interacting residues, exclusive of the (p)ppGpp binding site, numbered from left to right according to the *S. coelicolor* IMPDH sequence (highlighted): Arg71, Asp118, Arg125 and Asn144. The green arrows indicate the key nucleotide interacting residues shared by the second canonical and the (p)ppGpp sites: Glu 188, Lys206 and Lys210.

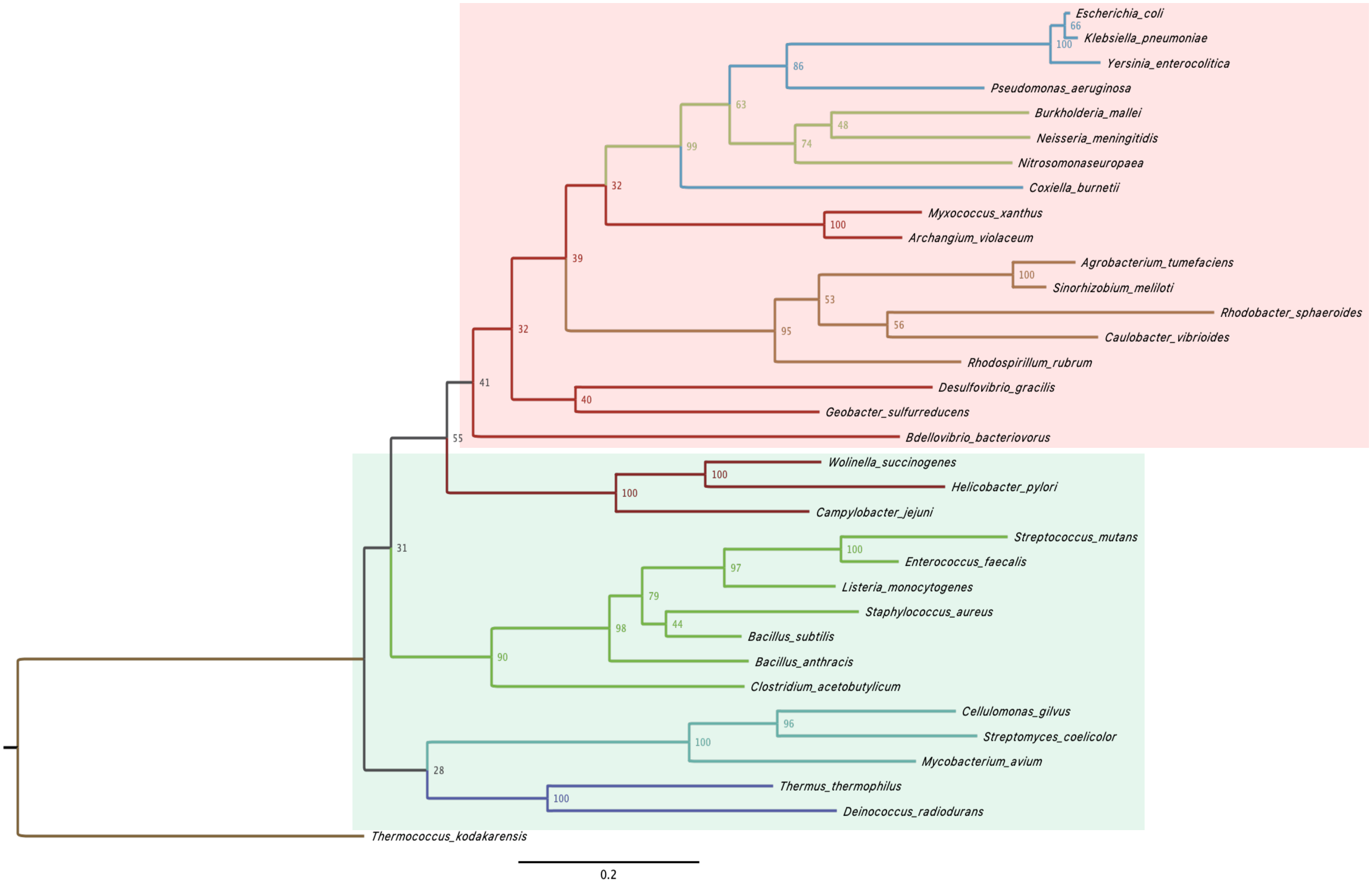

Supplemental Figure 9. *Evolutionary analysis of bacterial IMPDHs.* Rooted phylogenetic tree of bacterial IMPDHs (best model LG+G+I, (Le and Gascuel, 2008)). Branches are colored according to the taxonomical classification. Archaeal sequences serve as an outgroup. The length of the radius branches indicates the amount of change (substitutions/site) between a pair of nodes. The IMPDH enzymes that do and don´t contain the (p)ppGpp binding site are indicated in light green and red boxes, respectively.

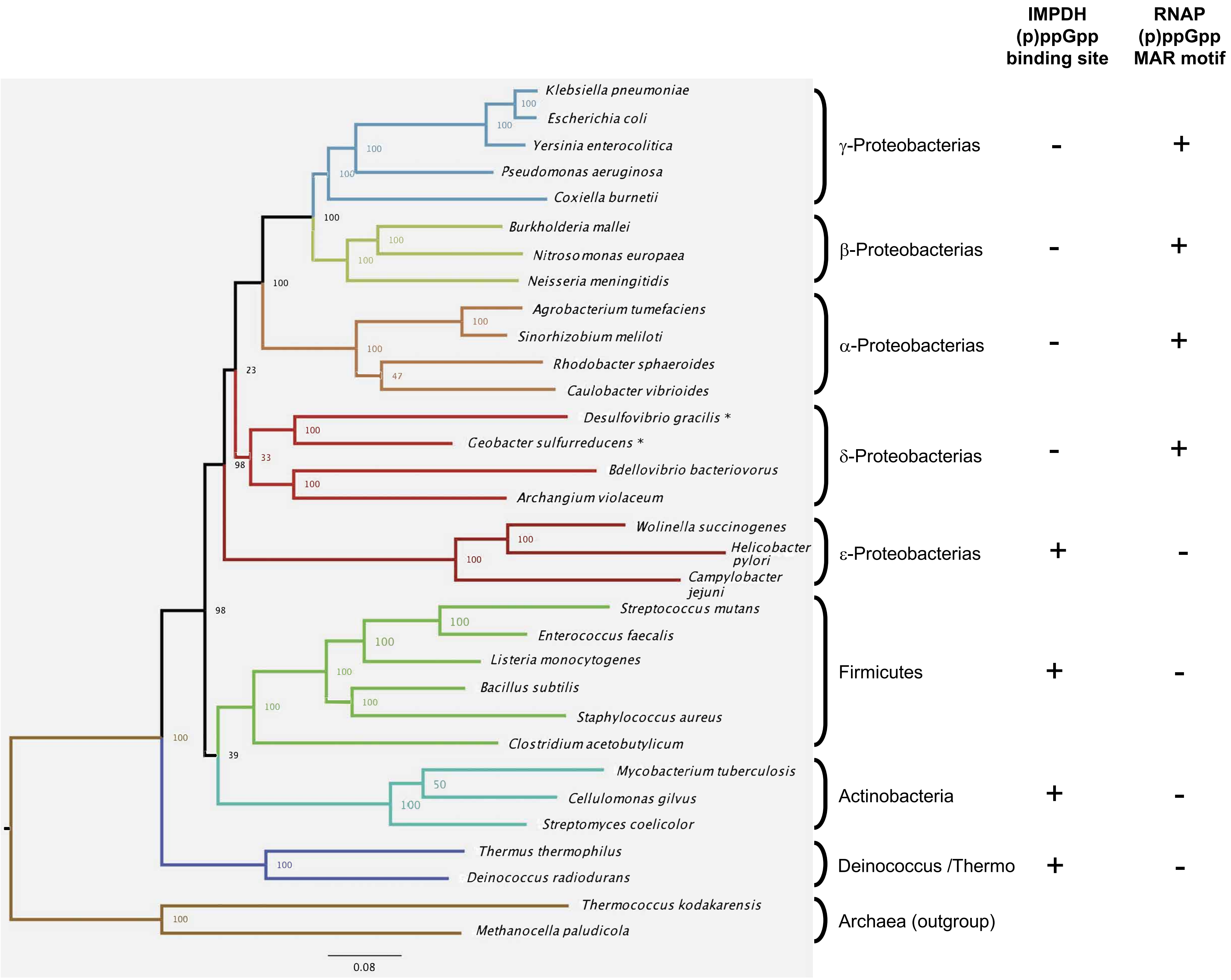

Supplemental Figure 10. *Tree of life of selected organisms*. Rooted phylogenetic tree of life constructed by the neighbor-joining method, showing bootstrap values (best model LG+G+F, (Le and Gascuel, 2008)), and branches colored as in Supplemental figure 6. Presence or absence of the (p)ppGpp binding site in IMPDH and RNAP sensitivity to (p)ppGpp is indicated on the right, according to our results and to the presence of the MAR motif in the w-subunit (Hauryliuk et al. 2015. Nat Rev Microb 13, 298-309). α, β, γ and δ-Proteobacteria do not contain the (p)ppGpp binding site, in contrast to ε-Proteobacteria. Nonetheless, some δ-Proteobacteria IMPDHs (marked with an asterisk: *D. gracilis* and *G. sulfurreducens*) contain the (p)ppGpp binding site. Although the pattern observed in δ-Proteobacteria is unexpected, reassuringly, the species tree confidently groups all delta proteobacteria in a monophyletic clade. Thereby, it is possible that there has been a horizontal gene transfer into δ-Proteobacteria, but the low resolution in the phylogenetic tree of IMPDH, and the ambiguous positioning of δ-Proteobacteria within this tree, prevents drawing reliable conclusions. Alternatively, convergent evolution may be at play, with loss of (p)ppGpp sensitivity happening twice, once in the ancestor of α, β and γ-Proteobacteria and once within δ-Proteobacteria.

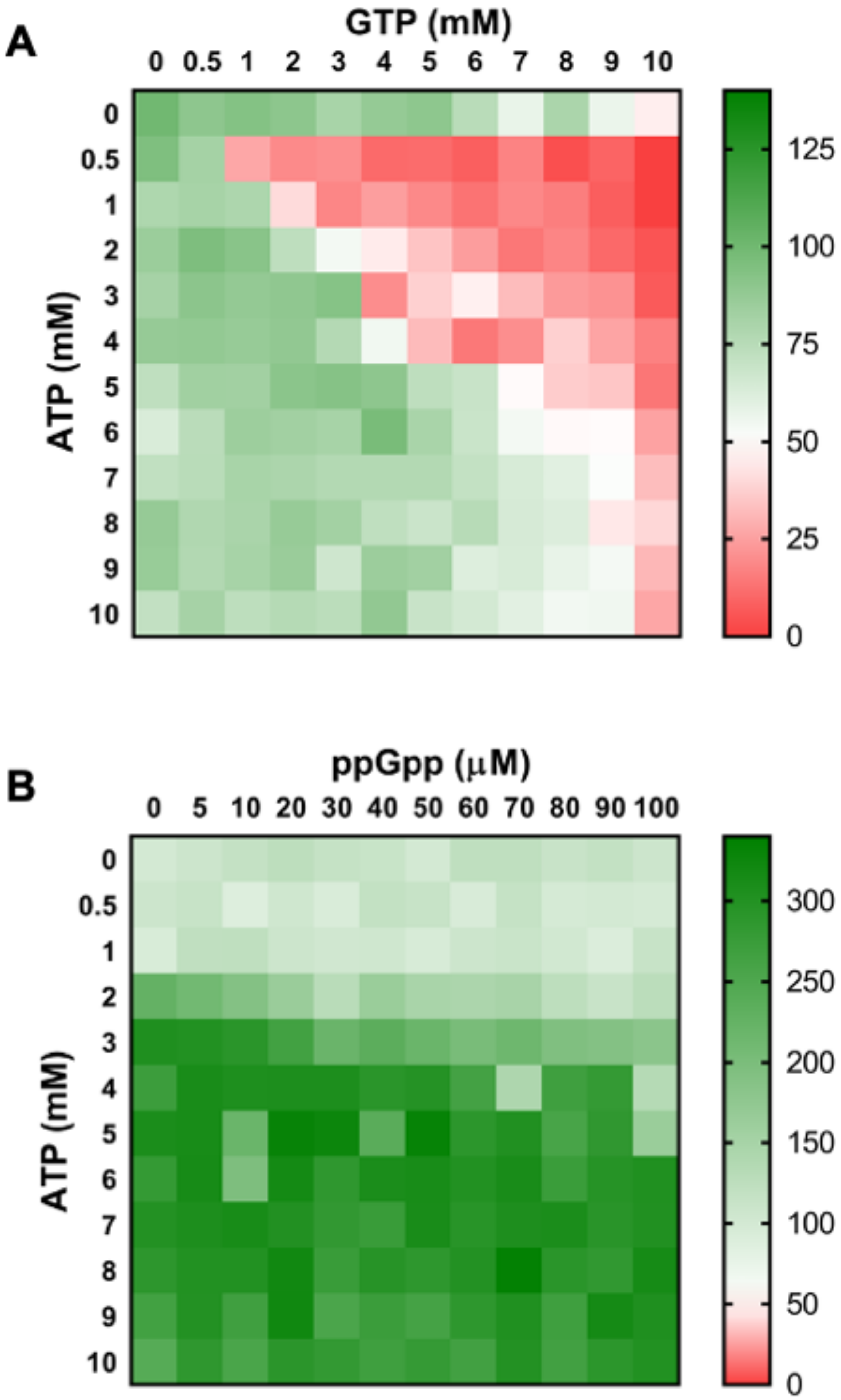

Supplemental Figure 11. *Lysine acetylation fine-tune bacterial IMPDH allosteric regulation in vitro.* Heatmap representation of the enzymatic percent activity. V_0 values normalized to the V_0 values in the absence of nucleotide of lysine acetylation mimetic mutants EcIMPDH-K203Q (**A**) and BsIMPDH-K206Q (**B**) at different ATP versus ppGpp or GTP concentrations. The V_o values used for normalization are 7.8 and 9.3 nM/sec for EcIMPDH-K203Q and BsIMPDH-K206Q, respectively.

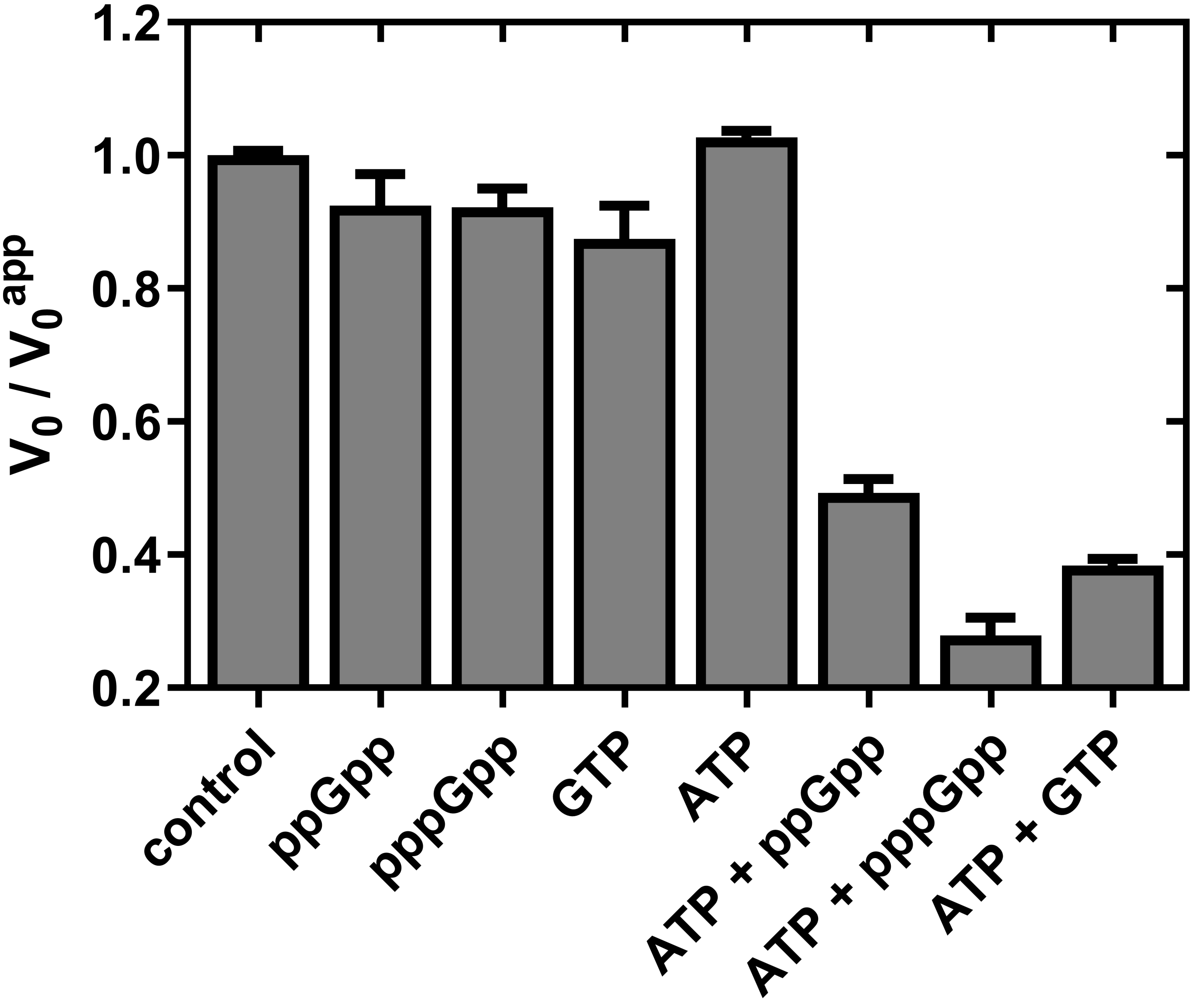

Supplemental Figure 12. *Effects of guanine nucleotides on the catalytic activity of HpIMPDH in vitro*. Normalized initial velocity (V_0) values calculated as the V_0 in the absence of nucleotides divided by the corresponding values in the presence of the indicated ones. The experiment was performed at 32ºC in buffer 100 mM Tris-HCl, pH 8.0, 100 mM KCl, 1 mM DTT, 1 mM free $MgCl_2$, 50 nM enzyme, 0.5 mM NAD^+ and 0.5 mM IMP. Nucleotide concentrations were (from left to right): 0.5 mM (p)ppGpp, 4 mM GTP, 2 mM ATP, 0.25 mM ATP + 0.5 mM (p)ppGpp, 0.25 mM ATP + 4 mM GTP. The V_o value used for normalization was 6.83 ± 0.1 nM/sec.
